# Frequency Dependence of Signal Power and Spatial Reach of the Local Field Potential

**DOI:** 10.1371/journal.pcbi.1003137

**Published:** 2013-07-18

**Authors:** Szymon Łęski, Henrik Lindén, Tom Tetzlaff, Klas H. Pettersen, Gaute T. Einevoll

**Affiliations:** 1 Department of Mathematical Sciences and Technology, Norwegian University of Life Sciences, Ås, Norway; 2 Department of Neurophysiology, Nencki Institute of Experimental Biology, Warsaw, Poland; 3 Department of Computational Biology, School of Computer Science and Communication, Royal Institute of Technology (KTH), Stockholm, Sweden; 4 Institute of Neuroscience and Medicine (INM-6) & Institute for Advanced Simulation (IAS-6), Jülich Research Centre and JARA, Jülich, Germany; Indiana University, United States of America

## Abstract

Despite its century-old use, the interpretation of local field potentials (LFPs), the low-frequency part of electrical signals recorded in the brain, is still debated. In cortex the LFP appears to mainly stem from transmembrane neuronal currents following synaptic input, and obvious questions regarding the ‘locality’ of the LFP are: What is the size of the signal-generating region, i.e., the spatial reach, around a recording contact? How far does the LFP signal extend outside a synaptically activated neuronal population? And how do the answers depend on the temporal frequency of the LFP signal? Experimental inquiries have given conflicting results, and we here pursue a modeling approach based on a well-established biophysical forward-modeling scheme incorporating detailed reconstructed neuronal morphologies in precise calculations of population LFPs including thousands of neurons. The two key factors determining the frequency dependence of LFP are the spatial decay of the single-neuron LFP contribution and the conversion of synaptic input correlations into correlations between single-neuron LFP contributions. Both factors are seen to give low-pass filtering of the LFP signal power. For uncorrelated input only the first factor is relevant, and here a modest reduction (<50%) in the spatial reach is observed for higher frequencies (>100 Hz) compared to the near-DC (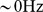) value of about 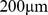. Much larger frequency-dependent effects are seen when populations of pyramidal neurons receive correlated and spatially asymmetric inputs: the low-frequency (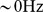) LFP power can here be an order of magnitude or more larger than at 60 Hz. Moreover, the low-frequency LFP components have larger spatial reach and extend further outside the active population than high-frequency components. Further, the spatial LFP profiles for such populations typically span the full vertical extent of the dendrites of neurons in the population. Our numerical findings are backed up by an intuitive simplified model for the generation of population LFP.

## Author Summary

The first recording of electrical potential from brain activity was reported already in 1875, but still the interpretation of the signal is debated. To take full advantage of the new generation of microelectrodes with hundreds or even thousands of electrode contacts, an accurate quantitative link between what is measured and the underlying neural circuit activity is needed. Here we address the question of how the observed frequency dependence of recorded local field potentials (LFPs) should be interpreted. By use of a well-established biophysical modeling scheme, combined with detailed reconstructed neuronal morphologies, we find that correlations in the synaptic inputs onto a population of pyramidal cells may significantly boost the low-frequency components and affect the spatial profile of the generated LFP. We further find that these low-frequency components may be less ‘local’ than the high-frequency LFP components in the sense that (1) the size of signal-generation region of the LFP recorded at an electrode is larger and (2) the LFP generated by a synaptically activated population spreads further outside the population edge due to volume conduction.

## Introduction

The measurement of electrical potentials in the brain has a more than hundred year old history [1]. While the high-frequency part has been successfully used as a measure of spiking activity in a handful of surrounding neurons, the interpretation of the low-frequency part, the local field potential (LFP), has proved more difficult. Current-source density (CSD) analysis of multisite LFP recordings across well-organized layered neural structures such as cortex and hippocampus, was introduced in the 1950's [2]. However, even if the CSD is a more local measure of neural activity than the LFP [3]–[8], the interpretation in terms of underlying activity in neural populations is inherently ambiguous [9], [10]. Thus in many in vivo applications, for example when investigating receptive fields in sensory systems, the LFP signal was discarded altogether. The LFP signal has seen a revival in the last decade, however. This is due to the rapid development of new silicon-based microelectrodes now allowing for simultaneous recordings of LFP at tens or hundreds of contacts [11]–[14] (and availability of affordable computer storage), the realization among neuroscientists that the LFP offers a unique window into neural activity at the population level [9], [15]–[23], and the possibility of using the LFP signal in brain-machine interfaces [24]–[27].

To take full advantage of the opportunities offered by this new recording technology, a precise understanding of the link between the recorded LFP and the underlying neural activity is required. For example, two obvious questions regarding the ‘locality’ of the LFP that need quantitative answers are: (1) What is the size of the signal-generating region, i.e., spatial reach, around a recording contact? (2) How far does the LFP signal extend outside an active population due to volume conduction? The first question has been addressed in several experimental studies, with resulting estimates for the spatial reach in cortex varying from a few hundred micrometers to several millimeters [28]–[33]. This large range in reported experimental estimates presumably reflects that the spatial reach depends strongly on the spatiotemporal properties of the underlying spiking network activity, in particular the level of correlations [34]. These critical network features will not only vary between the different brain regions and species studied, but also depend on the brain state.

In cortex, thousands of neurons contribute to the LFP, making the signal inherently difficult to interpret. Fortunately, the ‘measurement physics’, i.e., the biophysical link between neural activity and what is measured, is well understood: According to well-established volume-conductor theory [10], [35], the recorded LFPs stem from appropriately weighted contributions from transmembrane currents in the vicinity of the electrode contact. Building on pioneering work by Rall in the 1960's [35], [36], a forward-modeling scheme incorporating detailed reconstructed neuronal morphologies in precise calculations of extracellular potentials has been established [37] and used to explore both spikes [37]–[41] and LFPs [9], [34], [41]–[43] generated by single neurons [37]–[40], [42] and neural populations [9], [34], [41]. Unlike in experiments, this modeling scheme allows for a clear separation between volume conduction effects and effects of spatiotemporal variations in spiking network activity in determining population LFPs. In [34] it was used in a thorough investigation of the locality of LFP. It was found that the size of the LFP-generating region depends on the neuron morphology, the synapse distribution and correlations in synaptic activity. For uncorrelated activity, the LFP represents neurons in a small region (that is, a few hundred micrometers around the electrode contact), while in the case of correlated input the size of the generating region is determined by the spatial range of correlated synaptic activity and could thus be much larger. Specifically, it was found that correlated synaptic inputs onto either the apical or basal dendrites of a population of pyramidal neurons could give orders of magnitude larger LFPs, and a much larger spatial reach, compared to the situations with (1) the same correlated input spread homogeneously over the neuronal dendrite or (2) similar uncorrelated synaptic inputs placed evenly or unevenly over the neurons.

As shown in [34], the relative contributions to the population LFP from neurons at different distances from the electrode will depend on three factors: First, the amplitude of the LFP generated by a single neuron decays with distance (typically as 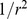 for distances beyond a few hundred micrometers, less sharply closer to the neuron). Thus single neurons close to the electrode will contribute more to the LFP than if it was placed further away. Second, for a disc-like population, characteristic for a laminar population in a cortical column, it follows that with constant neuron density, the number of neurons located on a ring at a particular radial distance 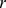 from the electrode will increase linearly with 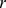. Third, with correlated synaptic inputs onto a neural population, the LFP contributions from different cells will also become correlated, or synchronized, and will effectively boost the contributions to the LFP. The contributions from different rings of neurons will thus be determined by the interplay of these three factors. In [34] a simplified model for LFP generation based on these elements, (1) the decay of the single-neuron contribution with the distance from the electrode, (2) the population geometry, and (3) the correlation of LFP contributions from individual neural sources, was constructed. We found this simple model to not only give qualitative insight into the generation of population LFPs, but also quantitatively accurate predictions of the size of the signal-generating region and the decay of the signal outside an active population. Here we extend this work by examining the frequency dependence of the LFP.

Strong frequency dependencies have been observed both in the tuning properties [28], [29] and information content [18], [22] of cortical LFPs. For example, the low-frequency LFP (less than 12 Hz) has been shown to carry complementary information to the gamma-range LFP (30–100 Hz) in V1 of macaque monkeys during naturalistic visual stimulation [22]. To properly interpret such experiments, it is thus important to know how spatial reach of the LFP varies across frequencies and whether the biophysics of LFP signal generation boost some frequencies compared to others. The high-frequency LFP components are, for example, expected to be more local than the low-frequency components due to ‘intrinsic dendritic filtering’ [42], i.e. due to the reduction of the (effective) current-dipoles with increasing frequency resulting from the capacitive properties of the dendritic membrane [10].

In [34] we used the biophysical forward-modeling scheme to investigate the total population LFP, i.e., the total signal generated across all frequencies. Here we use the same scheme to investigate both the distribution of the power of synaptically generated LFP between different frequency bands and the frequency dependence of the locality of the LFP signal. In terms of the latter, we study the size of the signal-generating region (spatial reach) as well as the spatial extension of the LFP signal outside an active population — for each frequency component separately.

**Figure 1:**
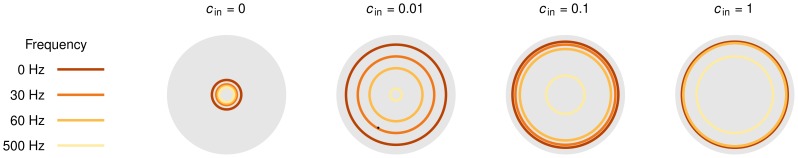
Spatial reach of different frequency components of LFP for different levels of synaptic input correlations 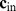
 **.** Color lines denote parts of the whole population (gray, radius = 1 mm) which contribute 95% of LFP amplitude at given frequency in the middle of the population, at the soma level. Results for layer-5 pyramidal cell with basal input.

We also use a frequency-resolved (i.e. dealing with each frequency component separately) version of the simplified model developed in [34] to guide our investigation of this frequency dependence. The population geometry (factor 2) does obviously not change with frequency. In contrast, the single-neuron LFP contribution (factor 1) decays faster with distance for higher LFP frequencies due to the intrinsic dendritic filtering effect [40], [42], but an equally important factor turns out to be the frequency dependence of the ‘correlation transfer’, i.e., how correlations in the synaptic input are transferred to correlations between the single-neuron LFP contributions (factor 3). As an example, Figure 1 illustrates how the frequency-resolved spatial reach varies with the input correlation for a pyramidal population receiving basal synaptic inputs. We show that when the frequency dependencies of factors 1 and 3 are incorporated, the simplified model can still account well for the results obtained by comprehensive numerical investigations. To allow for direct use of the simplified model in future applications, we here thus present and tabulate numerical results for the frequency dependence of these key factors for a variety of situations.

Note that we here for simplicity will refer to all calculated extracellular potentials as ‘LFPs’ even if we consider frequencies as high as 500 Hz which sometimes are regarded to be outside the LFP band. Further, spikes, that is, the extracellular signatures of action potentials, may contribute to recorded extracellular potentials at frequencies as low as 100 Hz [40], [44]–[47]. While the intrinsic dendritic filtering effect [40] and correlations [44] also are critical in determining the contribution from spikes to the LFP, our focus here is on the direct contributions from synaptic inputs.

The paper is organized as follows: first we describe the biophysical model of LFP and our simulation setup, present the simplified model of the population LFP, and review its ingredients. Then we present detailed results of the simulations: we analyze the frequency content of the population LFP, the reach of different frequency components, the decay of the signal outside of the population, and the depth-dependence of the LFP. Next we discuss the implications of our results for interpretation of electrophysiological data in terms of the underlying neural activity. Finally, in Methods we give details of the simulation setup and the mathematical model.

## Results

### Biophysical origin of LFP

Extracellular potentials are generated by transmembrane currents [48]. In the commonly used *volume conductor theory*, also employed here, the extracellular medium is modeled as a smooth three-dimensional continuum with transmembrane currents representing *volume current sources*. The fundamental formula relating neural activity in an infinite volume conductor to the generation of the LFP 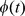 at a position 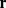 is given by [10], [37]

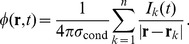
(1)Here 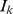 denotes the transmembrane current (including the capacitive current) in a neural compartment 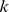 positioned at 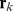, and the extracellular conductivity, here assumed real (ohmic), isotropic (same in all directions) and homogeneous (same at all positions), is denoted by 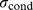.

A key feature of Equation 1 is that it is linear, i.e., the contributions to the LFP from the various compartments in a neuron sum up. Likewise the contributions from all the neurons in a population add up linearly.

The transmembrane currents 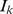 setting up the extracellular potentials according to Equation 1 are calculated by means of standard multicompartmental modeling techniques, here by use of the simulation tool NEURON [49].

### Simulations of LFP

**Figure 2:**
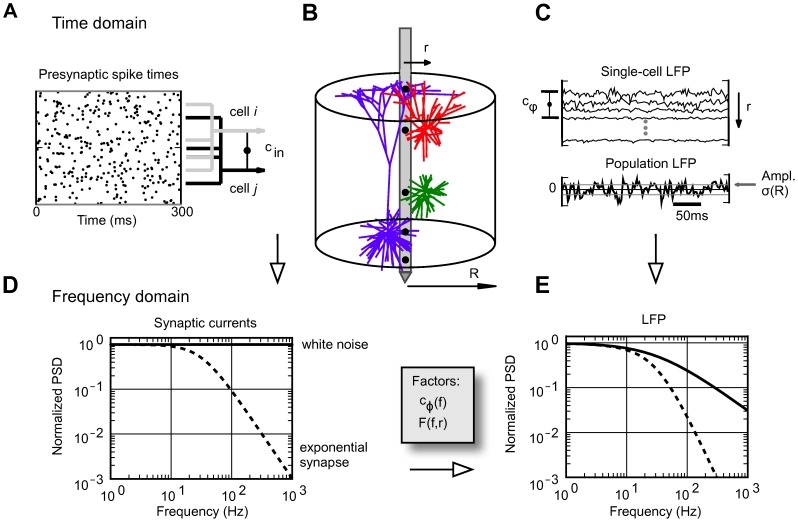
Simulation setup. A. Input spike trains are either generated independently for each cell (uncorrelated input), or chosen from a common pool (correlated input: every two cells share a fraction 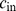 of inputs). B. Model cells (red: L3 pyramidal cell, green: L4 stellate cell, blue: L5 pyramidal cell) are placed with constant planar density 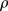 on a disc of radius 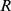, in this example with the recording electrode at the population center. Electrode positions shown as black dots. C. The population LFP is a sum of contributions from cells at different distances 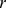. The dependence of the amplitude 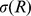 of the population LFP on the population radius 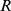 serves to define the spatial reach (see text). The correlations between inputs give raise to correlations 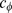 between single-cell LFP contributions. D. The synapses used in simulations yield a flat power spectrum of input current, but because of the frequency-dependence of single-cell *shape functions* 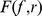 and *population-averaged coherence* 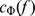 (see text), the resulting power spectrum of the population LFP is not flat (E). This LFP filtering effect will be present for any synapse type, such as the exponential synapse which in addition yields non-flat power spectrum of the input current (dashed curves in D, E).

An essential part of the present work is the numerical simulation of the LFP in the center of a disc-like population of cortical cells. The simulation setup is illustrated in Figure 2. We consider a population of 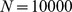 cells distributed homogeneously on a planar disc with a radius of 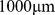, Figure 2B. The number of cells is chosen to be the same as in [34] and translates to the planar cell density 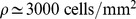 for each population. This density allows for efficient simulations and seems biologically plausible: a total planar density of, say, 50000 cortical neurons per 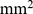 [50] divided by the number of relevant subpopulations (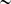
5–10), and finally multiplied by the fraction of neurons in the subpopulation receiving synaptic inputs, will give on the order of a few thousand single-neuron LFP sources per 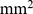.

The somas of the cells are all positioned at the same depth, and the LFP is calculated at various ‘virtual electrode’ positions inside and outside the population. In this setup we investigate how the LFP signal increases as contributions from more and more distant neurons are included, i.e., we study how the root mean square amplitude 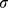 of the population LFP (obtained as a sum of single-cell contributions) depends on the radius 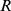 of the subpopulation of cells included in the sum (Figure 2C).

In the simulations we use three different morphologically-detailed cell models shown in Figure 2B: the layer-3 and layer-5 pyramidal cells, and the layer-4 stellate cells. All neuron models are passive, i.e., without active conductances, and the extracellular signatures of action potentials (spikes) are thus not included. In combination with the use of current-based synapses (see next paragraph) this assumption makes the system linear so that each frequency (Fourier) component can be investigated separately. For each class of pyramidal cells we consider three different spatial patterns of synaptic input: the synapses are placed either in the apical region only, in the basal region only, or evenly over the whole cell. For the layer-4 stellate cells we consider only spatially homogeneous synaptic input, as these cells lack clearly defined dendritic regions. Each synapse is activated with a Poissonian spike train, the spike trains can be either generated independently for each cell, or chosen from a common pool to model input correlations, Figure 2A.

The synaptic currents are modeled as 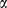-functions with a very short time constant (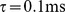) to assure that no frequency filtering is imposed by the synapses themselves. In the frequency range considered in the present simulations (up to 500 Hz) each synaptic input current thus effectively corresponds to a 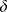-function with a white (flat) power spectrum. With Poissonian spike statistics, which also implies a white power spectrum, the power spectrum of the input current is flat, Figure 2D. Hence the only frequency filtering in our simulation setup will come from the intrinsic dendritic filtering effect [40], [42] due to electrical properties of the cable and the summation of the single-neuron LFP contributions to form the population LFP, Figure 2E. If any frequency filtering was to be imposed by the synapse, such as the exponential synapse (Figure 2D), the power spectra of the population LFP would be determined by the superposition of the synaptic and dendritic filters, Figure 2E, i.e., by multiplying the transfer functions of the two filters. For further details on the simulations we refer to the Methods section.

### Simplified model of population LFP

To understand how the population signal emerges from single-cell contributions we use a simplified mathematical model, which is a frequency-resolved version of the model introduced in [34].

We assume that the *power spectral density* (PSD) of the contribution to the LFP from the *i*-th cell at given frequency can be factorized as
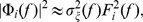
(2)where 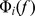 is the Fourier transform of the single-cell LFP 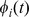, |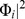 is the PSD of the single-cell LFP, 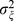 is the PSD of the synaptic input current, and 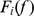 is the frequency-dependent *shape function* of the *i*-th cell, which carries the information about how the root mean square amplitude of the signal at given frequency decays with distance at a given depth. Moreover, we assume that the shape function of each cell in the population can be replaced with a single, distance- and frequency-dependent function:
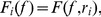
(3)that is, we assume that the shape function 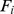 only depends on the frequency and the lateral distance 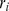 from the recording electrode (Figure 2B), and neglect variation in the single-neuron LFP contributions due to other factors. For each particular morphology (layer-3/layer-4/layer-5) and synaptic stimulation pattern (homogeneous/apical/basal), the LFP contribution from each cell in the population is thus described with the function 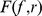. Note that for the special case of white-noise input (i.e., 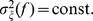), the squared shape function 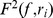 will be proportional to the PSD of the single-cell contribution to the LFP.

The summation of single-cell LFPs to the population signal depends on the correlation between the single-cell LFP contributions. In the case of *uncorrelated* input this amounts to simply adding the variances of the single-cell LFPs. For a disc-like population of radius 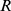 we thus obtain the following expression for the PSD of the signal at the center:
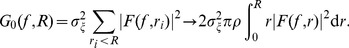
(4)On the other hand, if the single-cell LFPs are *fully correlated* (identical), the PSD of the signal is found by adding the single amplitudes, not variances, and we thus obtain
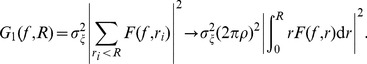
(5)


In our simulation setup the single-cell LFP contributions from two equidistant neurons (i.e., same 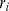) are not identical even for the maximum level of input correlations 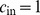: while the same spike trains are used to synaptically stimulate the cell, they will not in general activate an identical set of synapses (see Methods). Moreover, as we now work in the frequency domain, the correlation between single-cell contributions to the LFP (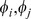) is naturally replaced by their *coherence* (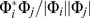), which, in general, depends on the frequency.

If we approximate the LFP coherence between each pair of cells by the population-averaged LFP coherence 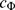, then the PSD is given by
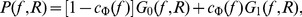
(6)where 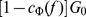 is the contribution resulting from uncorrelated inputs, and 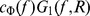 represents the contribution of correlated inputs (see Methods for the full derivation of this formula). Note that the root mean square amplitude 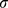 of the signal (see Figure 2) is related to the PSD 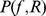 through
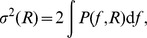
where the integration is between 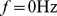 and 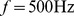 (half the sampling frequency).

### Illustration of use of simplified model of population LFP

**Figure 3:**
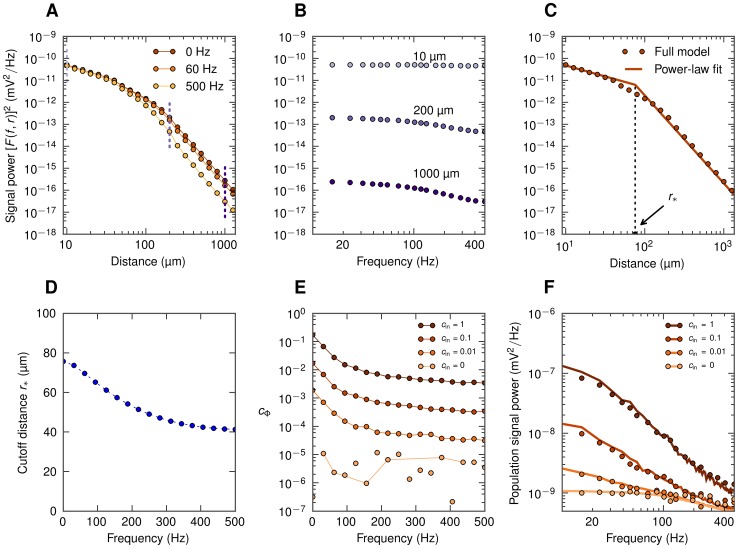
Ingredients of the simplified LFP model for soma-level LFP for layer-5 cell with basal synaptic input. A. Spatial decay in lateral direction for the squared single-cell shape functions 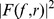 for three different frequencies 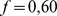 and 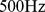. B. Single-cell LFP spectra 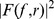 for three different lateral distances from the soma (dotted vertical lines in A). C. Log-log plot of the squared near-DC (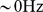) shape function 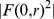 (dots) approximated by a piecewise-linear function with cutoff distance 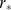 (line; see Eq. 7). D. Frequency dependence of the cutoff distance 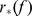. E. Population-averaged LFP coherence 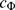 for different input correlation levels 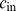. Dots not connected with lines indicate that 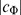 is plotted in place of spurious negative values (see Methods). F. Power spectra 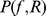 of the compound LFP (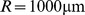); dots correspond to simulation; lines correspond to predictions from simplified model, Eq. 6, based on 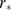 and 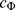 given in D and E, respectively.

Before embarking on the comprehensive numerical evaluation of the ingredients of the simplified model in the next Section and its use in the remainder of the Results, we illustrate in Figure 3 the key features of the model on a specific example, a population of layer-5 cells receiving basal synaptic inputs.

The first ingredient that must be determined is the shape function 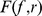 in Equation 3. Figures 3A and B show the numerically evaluated squared shape functions 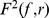 at the soma level as a function of distance from the neuron (for three selected frequency bands) and frequency (for three distances), respectively. Figure 3C illustrates the fitting of the numerical results (full model) to a piecewise power-law expression (see Equation 7 below) for 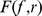. The fitted values of the key parameter in this power-law function, the cutoff distance 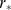, are found to depend on frequency reflecting the intrinsic dendritic filtering effect (Figure 3D). The second ingredient is the average coherence 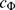 between single-neuron LFP contributions. The numerically evaluated 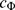, shown in Figure 3E for four values of the input correlation 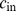, is seen to depend even more strongly on frequency.

Next we can plug 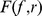 into the integrals, Equations 4 and 5, to obtain 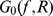 and 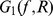, respectively. Finally, the population LFP power is evaluated by combining 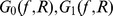, and 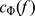 in Equation 6. The results for the present example are displayed in Figure 3F. As observed, correlated input boosts the low-frequency population LFP up to two orders of magnitude, a key feature which is seen both in the numerical simulations (dots) and in the simplified model (solid lines).

The population LFP shown in Figure 3F is measured at the center of a population with radius 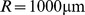. In the next sections we investigate how the LFP amplitude depends on the various factors and also investigate how local the LFP is in the various situations: First, the size of the signal-generating region is probed by studying how the LFP amplitude measured at the soma level grows when the population radius 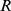 is increased. From this a measure of the *spatial reach* can be extracted. Next, we investigate how the measured LFP power decays when the electrode is moved outside the active population. Finally, we investigate the depth-resolved LFP profile, i.e., the locality of the LFP changes in the vertical direction.

### Numerical evaluation of ingredients of simplified model

Equation 6 implies that any frequency dependence of the population LFP (for example, frequency dependence of the spatial reach) in general will result from the interplay of two separate effects: (1) frequency dependence of the single-cell shape functions 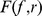 and (2) frequency dependence of the coherence 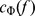 between single-cell contributions to the population signal. These two effects are addressed next.

#### Frequency dependence of shape function

The power of the extracellular potential from a single neuron decays when we move away from the cell, and the rate of the decay depends on the frequency of the signal. In Figure 3A we have plotted squared shape functions 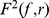 at the soma level for three selected frequency bands for the case with the layer-5 cell receiving basal synaptic stimulation. We observe that the high-frequency LFP component decays faster with distance than the low-frequency component. This leads to the low-pass filtered power spectra seen in Figure 3B and is consistent with our previous observations of low-pass filtering in dendritic cables, i.e., the intrinsic dendritic filtering effect [40], [42]. To quantify this phenomenon we approximate the actual shape functions with simplified power-law shape functions with frequency-dependent parameters. Specifically, at the soma level the amplitude of the single-cell LFP is, following [51], modeled as:
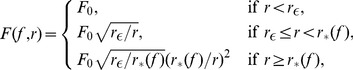
(7)i.e., the shape function is approximated by 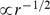 close to the cell (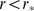) and by 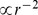 (dipole) in the far-field regime (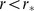). The constant value of 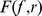 is used for 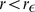 to avoid the unphysical divergence; however, in the numerical evaluation at the soma level 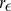 is effectively set to zero. The parameter 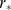 thus represents the *cutoff distance* where the LFP contribution switches from the near-field (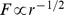) to the far-field regime (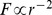), see fitted curve in Figure 3C. This parametric representation of the shape function allows us to express the functions 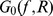 and 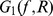 (Equations 4 and 5) explicitly in terms of the cutoff distance 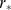, see Methods for details. The observed reduction of 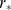 with increasing frequency (Figure 3D) is intimately related to the corresponding reduction of the frequency-dependent electrotonic length constant in dendrites [10], [40]. In the example shown in Figure 3A the transition to dipole decay occurs closer to the cell for the high-frequency signal (at about 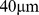) than for the low-frequency components (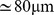).

**Figure 4:**
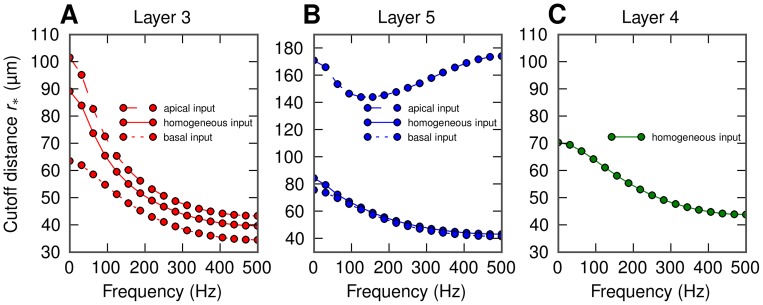
Frequency dependence of the cutoff distance 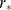 for soma-level LFP for all situations considered: homogeneous (solid), apical (dashed) and basal synaptic input (dotted) applied to the layer-3 pyramidal cell (A), the layer-5 pyramidal cell (B), and the layer-4 stellate cell (C). Cell morphologies depicted in Figure 2B. Dots in A, B, C represent the actual frequency resolution, thin lines serve to guide the eye.

In Figure 4 we show the calculated cutoff distance 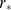 for LFPs at the soma level for the various situations considered in the present paper involving the layer-3 pyramidal neuron (4A), the layer-5 pyramidal neuron (4B), and the layer-4 stellate neuron (4C). For the pyramidal neurons we consider three spatial patterns of synaptic inputs, that is homogeneous, only apical or only basal [34]. All these combinations of cell morphology and stimulation pattern exhibit similar behavior as in our example (Figure 3): 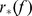 decays with increasing frequency. The only exception is the layer-5 cell with apical input, where 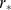 is very large, and also exhibits a minimum around 150 Hz. This reflects that the geometry of this situation is unique, with the synaptic input positioned far above the soma level where the LFP is recorded. As a consequence the shrinkage of the current dipole with increasing frequency will be accompanied by a vertical shift of the mean position of the current dipole in the apical direction. In this situation where the electrode is far below the effective current dipole, there will be little change in the signal when the lateral distance is changed (see Figure 2D in [34]). This will translate to a larger value of 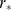 with our current fitting procedure. The squared shape functions and the single-cell power spectra for the remaining situations (all apart from layer-5 cell with basal synaptic input) are shown in Figures S2A, B to S7A, B.

#### Frequency dependence of coherence

The single-cell shape functions 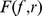 alone are generally not sufficient to predict the population LFP. The missing component is 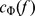, the frequency-dependent population-averaged coherence between single-cell LFP contributions. This quantity can be estimated from population simulations, as described in detail in Methods, Equation 17. Coherence curves for different input correlation levels for our example (LFP recorded at the soma level at the center of a layer-5 cell population receiving basal stimulation) are shown in Figure 3E. The coherence 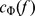 is seen to be higher for low-frequency components. This may be understood on biophysical grounds by considering the dendritic morphology of the cell: for high-frequency synaptic input the return currents will be closer along the dendrite to the synaptic currents because of filtering in the dendritic cable [40]. For the example in Figure 3E with basal stimulation of layer-5 pyramidal neurons, the resulting current dipoles will be aligned along the short basal dendritic segments, which converge at the soma from all angles. However, for low-frequency input some of the synaptic input current will return through the apical dendrite [42], and the orientation of the effective current dipoles will be more similar between cells, leading to a higher coherence.

By combining the shape functions 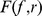 with the LFP coherence 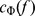 in the simplified model (Equation 6) we can now obtain predictions for the population LFP. The resulting PSD for our example situation is shown in Figure 3F and is seen to be in excellent agreement with the simulation results (see Figures S2C–S7C for the results for the remaining combinations of cell type and synaptic input patterns).

**Figure 5:**
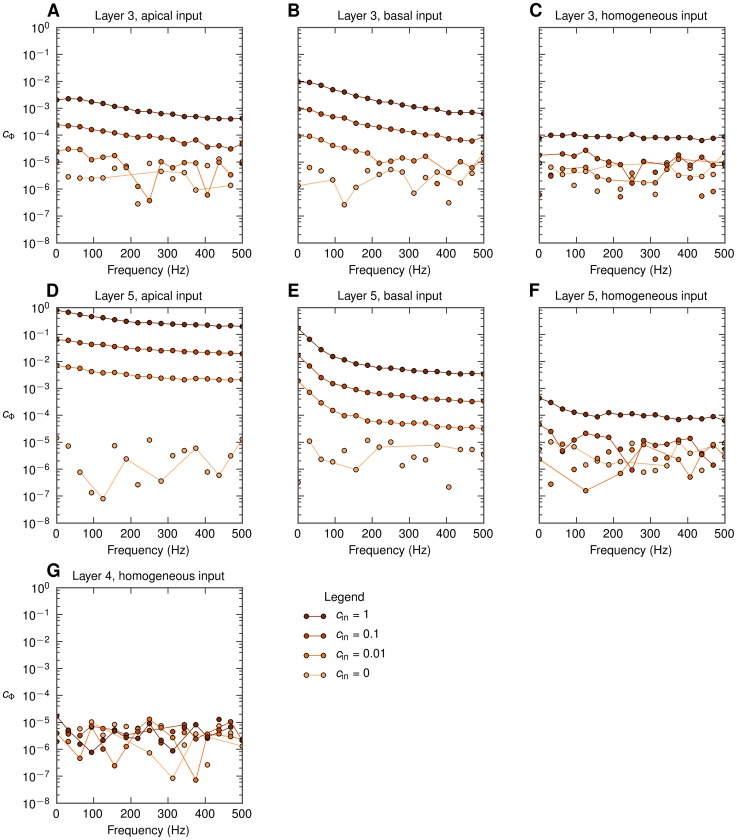
Frequency dependence of the population-averaged LFP coherence 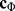 for soma-level LFP for all situations considered. Dots represent the actual frequency resolution, thin lines serve to guide the eye. Dots not connected with lines indicate that 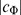 is plotted, see Methods. A, B, C: population of layer-3 cells; D, E, F: population of layer-5 cells, G: population of layer-4 cells; A, D: apical synaptic input; B, E: basal synaptic input; C, F, G: homogeneous synaptic input.

In Figure 5 we show the frequency dependence of the coherence 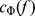 (measured at the soma level at the center of the populations) for the same full set of seven situations as depicted in Figure 4. A first observation is that for pyramidal neurons (layer-3, layer-5) with asymmetric synaptic input (either only apical or only basal), decay of 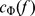 with increasing frequency is observed for all non-zero levels of input correlations 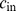. This low-pass filtering effect is seen to be strongest for the layer-5 cell with basal input (Figure 5A, 5B, 5D, 5E). However, when the same pyramidal neurons receive homogeneous synaptic inputs, the filtering effect is almost absent (Figure 5C, 5F). In that respect it resembles the situation with the stellate layer-4 cells receiving homogeneous synaptic input (Figure 5G) where 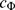 is essentially zero, implying that the correlations in the synaptic input do not translate into correlations of the single-neuron LFP contributions.

### Population LFP and spatial reach

**Figure 6:**
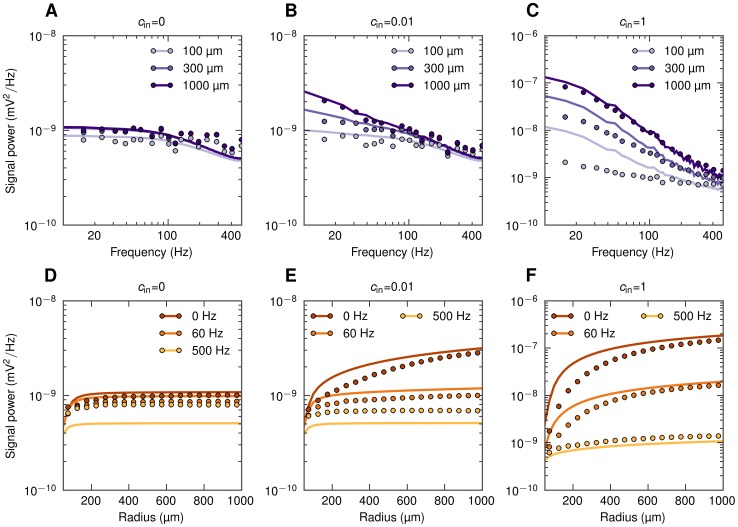
Power spectral density of population LFP at the soma level as a function of frequency and the population radius. Full simulation results (dots) and simplified model predictions (lines) for the LFP at the center of disc-like populations of layer-5 pyramidal cells receiving basal synaptic input. Three different input correlation levels 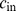 are considered. A, B, C: PSD of population LFP for three population radii 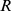. D, E, F: dependence of power of three different frequency components on the population radius 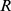.

As a first step towards exploring the spatial reach of the extracellular potential in our disc-like setup we next show how the population signal emerges from single-cell contributions and investigate frequency-related effects. In Figure 6 we present results both from the full simulation and the simplified model (Equation 6) for our example situation with the population of layer-5 cells receiving basal synaptic input.

In Figure 6A we show the PSD of the LFP produced by differently-sized populations of cells receiving uncorrelated synaptic input. While we observe some low-pass filtering (especially above 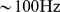) for all population sizes, the effect is not particularly strong. Figure 6D instead shows the PSD for the same uncorrelated situation as a function of the population radius 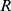. We observe that the LFP in all frequency bands saturates rather quickly with increasing population size, that is for 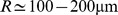. This implies that the contributions from uncorrelated neuronal LFP sources positioned more than a few hundred micrometers away from the electrode are negligible for all frequencies considered.

The situation changes dramatically for the case of correlated synaptic input (Figure 6B, 6C, 6E, 6F), both in terms of amplitude and frequency dependence. For the case with the maximum input correlations 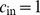 (Figure 6C, 6F), we see that the low-frequency power is up to two orders of magnitude larger than for the corresponding uncorrelated case. Further, a significant low-pass filtering effect is seen. For example, the low-frequency power (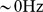) is an order of magnitude larger than the power at 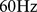 for 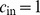 (Figure 6F). Another observation is that the low-frequency power grows much faster with increasing population radius than the high-frequency power (Figure 6E, 6F). Finally, the power of the population signal no longer seems to saturate as the population radius increases [34].

The predictions from the simplified model agree qualitatively with the full simulation results; however, we observe some clear deviations: First, in Figure 6D–F we see that the simplified model overestimates the power of the low-frequency components (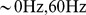). This is because the model here uses the approximate power-law shape functions (Equation 7) which lie above the numerically evaluated shape functions for low frequencies (Figure 3C). For high-frequency components (500 Hz), on the other hand, the opposite situation occurs (results for fitted approximate power-law function not shown). Second, in case of correlated input the model works better for the larger populations than for smaller ones. This is as expected given the present procedure for calculating the LFP coherence 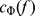 used in the simplified model: here this LFP coherence 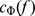 was extracted from the full population (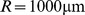) simulations, and the value obtained is not surprisingly a poor approximation when applied to populations which are much smaller. With 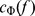 calculated for each population radius 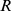 separately, the simplified model predictions significantly improve (Figure S1).

We are now ready to analyze the frequency dependence of the spatial reach of extracellular potential. Following [34] we define the *spatial reach* as the radius of the subpopulation which yields 95% of the root mean square amplitude in the population center compared to the largest population considered (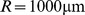). With this definition the spatial reach is easily found from the data presented in Figure 6D, 6E and 6F as the distance at which the amplitude of the LFP reaches 95% of the maximum value.

**Figure 7:**
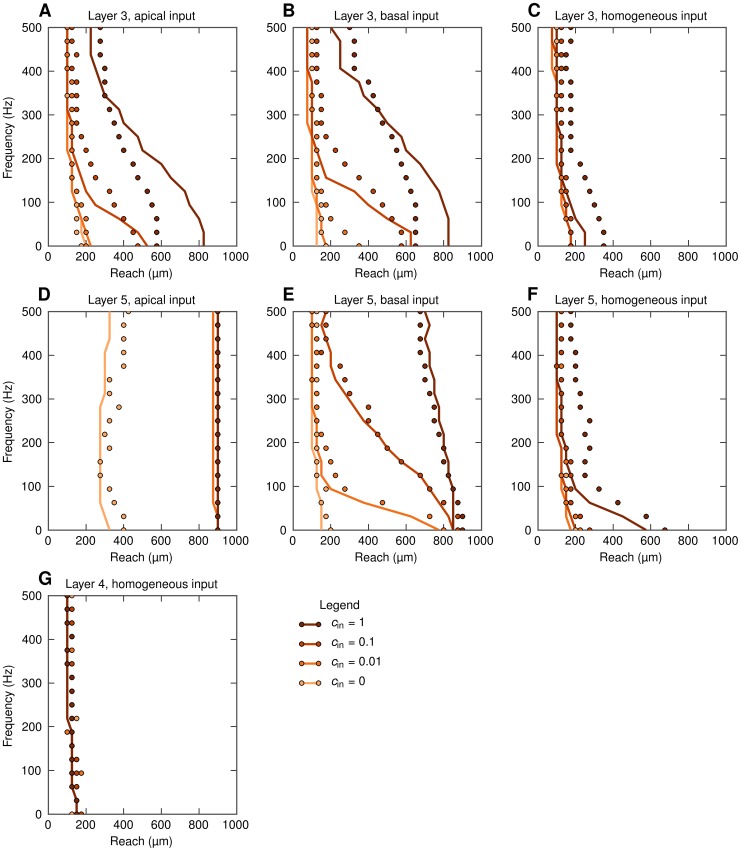
Spatial reach at soma level for different frequency components of LFP. Spatial reach is defined as the radius of a subpopulation contributing 95% of the root mean square amplitude of LFP compared to the amplitude for 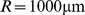. LFP is calculated at the soma level at the center of the population. Full simulation results plotted with dots; predictions from the simplified model (Equation 6) based on calculated values of 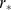 and 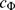 given in Figures 4 and 5, respectively, are shown with lines. A, B, C: population of layer-3 cells; D, E, F: population of layer-5 cells, G: population of layer-4 cells; A, D: apical synaptic input; B, E: basal synaptic input; C, F, G: homogeneous synaptic input.

The results for the spatial reach for all seven situations considered are shown in Figure 7. The reach is seen to vary both with the frequency 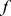 and the level of input correlation 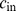, but the specific effects depend sensitively on the cell morphology and synaptic stimulation pattern. For the pyramidal cells with asymmetric input (either only basal or only apical) the spatial reach grows significantly with increasing input correlations 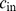 (Figure 7A, 7B, 7D, 7E). The effect is particularly prominent for lower frequencies, i.e., smaller levels of input correlations 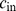 are needed to increase the spatial reach significantly. As a consequence, for certain correlation levels 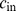 the spatial reach of the low-frequency components can differ a lot from the spatial reach of the high-frequency components. For example, in the situation with the layer-5 population receiving basal input with 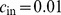, the spatial reach at 100 Hz is only around 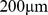, while the low-frequency reach is almost 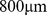. For the case of homogeneous inputs onto pyramidal neurons (Figure 7C, 7F) these effects are still present, but seen to be much weaker. For the layer-4 stellate cells the spatial reach is practically independent of the frequency 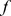 and the input correlation level 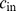, Figure 7G.

Note that the situation with the layer-5 population receiving only apical input is again somewhat different from the other cases. Here the spatial reach for the uncorrelated input is already quite large (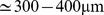) and the levels of the input correlation required to saturate the spatial reach at a maximum value possible in our setup are significantly smaller.

For the case of uncorrelated input we can obtain analytical expression for the spatial reach from the simplified model. Using Equations 4 and 7 we obtain an explicit formula for 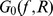 in terms of the cutoff distance 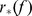 and the population radius 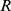, Equation 15. From this, we find in the limit of 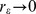, that the radius of the subpopulation contributing a fraction 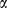 of the asymptotic amplitude (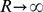) is equal to 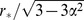 (valid for 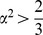). For our choice of 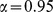 we find the spatial reach to be 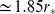.

### Lateral decay of LFP outside the population

The spatial reach we have discussed above represents an ‘electrode-centric’ point of view: we ask about the distance from the recording electrode of the neurons setting up the LFP signal. However, one can also take a ‘population-centric’ approach and instead ask how rapidly the LFP signal decays with distance outside an active population [34].

**Figure 8:**
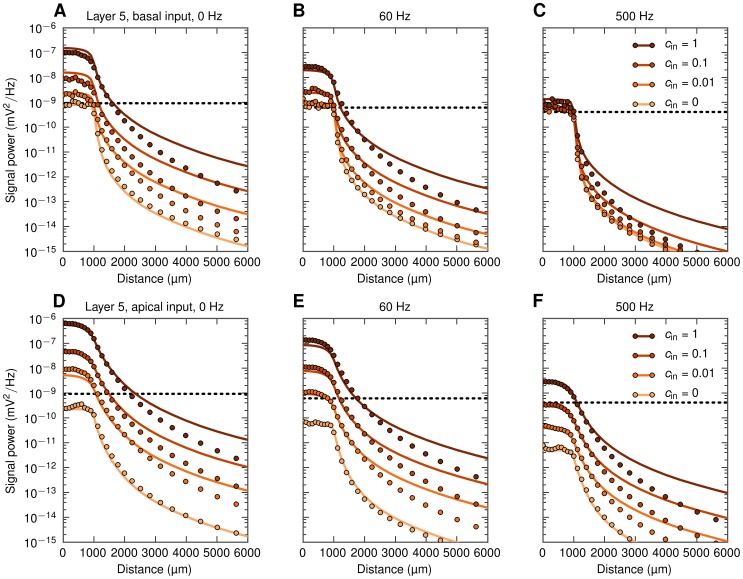
Decay of extracellular potential at the soma level outside populations of layer-5 cells with asymmetric input. Each of the panels shows full simulation results (dots) and predictions from simplified model, Equation 6 (lines) for one frequency band (0, 60, 500 Hz) and four input correlation levels. Horizontal dotted lines indicate ‘noise level’ (power of the signal generated by a population of uncorrelated cells with homogeneous input, see text). A, B, C: basal synaptic input. D, E, F: apical synaptic input.

In Figure 8 we show results for this situation, still with LFPs recorded at the soma level, for an example population (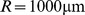) of layer-5 cells receiving basal or apical synaptic inputs. The first observation in the case of basal synaptic input is that the low- and medium-frequency LFP components (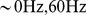) are significantly boosted, up to two orders of magnitude, by high levels of input correlations 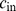 (Figure 8A, 8B). This applies both inside and outside of the population. For the high-frequency signal (500 Hz, Figure 8C), however, input correlations are seen to have only a small boosting effect on the signal amplitude. In the case of apical synaptic inputs the effect of increasing input correlations is seen to be more uniform across frequency bands, with the high-frequency components (500 Hz) being boosted by roughly the same factor as the low- and medium-frequency LFP components (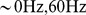), Figure 8D–8F.

The strong boosting of the LFP signal seen for correlated synaptic input for 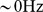 (Figure 8A) and 60 Hz (Figure 8B) has direct implications for how recorded LFP signals should be interpreted. As observed in these panels, the LFP measured a millimeter or more *outside* a highly-correlated populations can easily be larger than the LFP contribution from a similar, yet uncorrelated population surrounding the electrode. For the example, in Figure 8A we observe that the LFP signal recorded 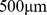 *outside* a correlated population with 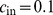 is still larger than the contribution recorded *inside* the same population receiving uncorrelated synaptic inputs (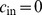). For 60 Hz (Figure 8B) the boosting effect is smaller, but still the signal recorded outside a correlated population may be larger than what is recorded inside an identical population receiving uncorrelated input. This dominance of LFPs from distant correlated populations over uncorrelated populations surrounding the electrode is seen to be even more pronounced for the apical-input case in the lower panels (Figure 8D–8F), further highlighting that the interpretation of the recorded LFPs in terms of activity in the neurons immediately surrounding the electrode has to be done with caution.

**Figure 9:**
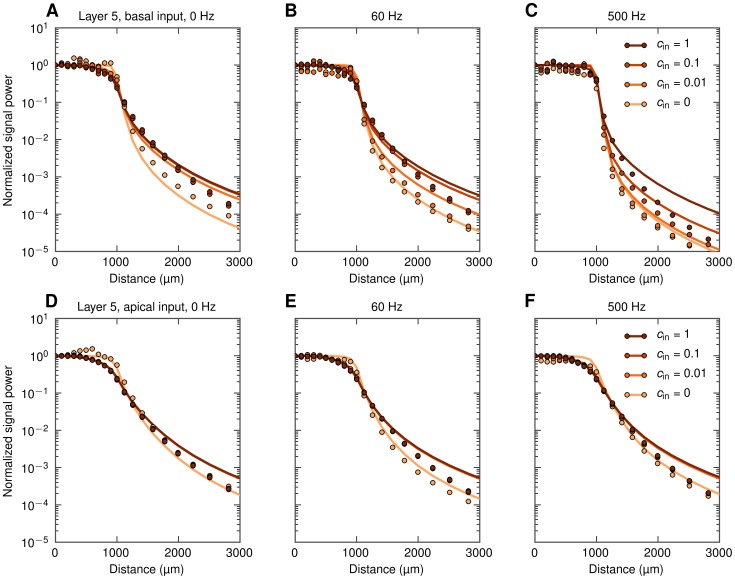
Decay of extracellular potential at the soma level outside populations of layer-5 cells with asymmetric input. Same as Figure 8, but with PSDs normalized to 1 at the population center, and the distance axis zoomed in to highlight the behavior around the edge of the population. A, B, C: basal synaptic input. D, E, F: apical synaptic input.

In Figure 9 we show the same PSDs as in Figure 8, but normalized to unity at the population center. This illustrates that the decay of the LFP is more abrupt around the population edge in the uncorrelated case than in correlated cases (this is especially prominent for the low-frequency components 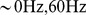). This is consistent with an observation made in [51] (see Figure 3.9 therein), namely that in the large-population limit the LFP signal power at the population edge will be reduced to half of power at the center for uncorrelated populations, while it will be reduced to a quarter of the center power for fully correlated populations. Here this difference between the correlated and uncorrelated cases is more pronounced for the low-frequency components, where the coherence 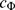 is largest.

In general, there are three key lengths determining the decay outside a population: the size of the population, the anatomical extension of the dendrites of the neurons, and the electrotonic length of the neuronal dendrites. In the examples depicted in Figures 8 and 9 we considered populations of layer-5 cells with a radius 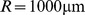. For smaller populations the abruptness of the decay outside the population edge will be less sharp as demonstrated in [51], but we refrain from a detailed study of the interplay of all these factors here.

**Figure 10:**
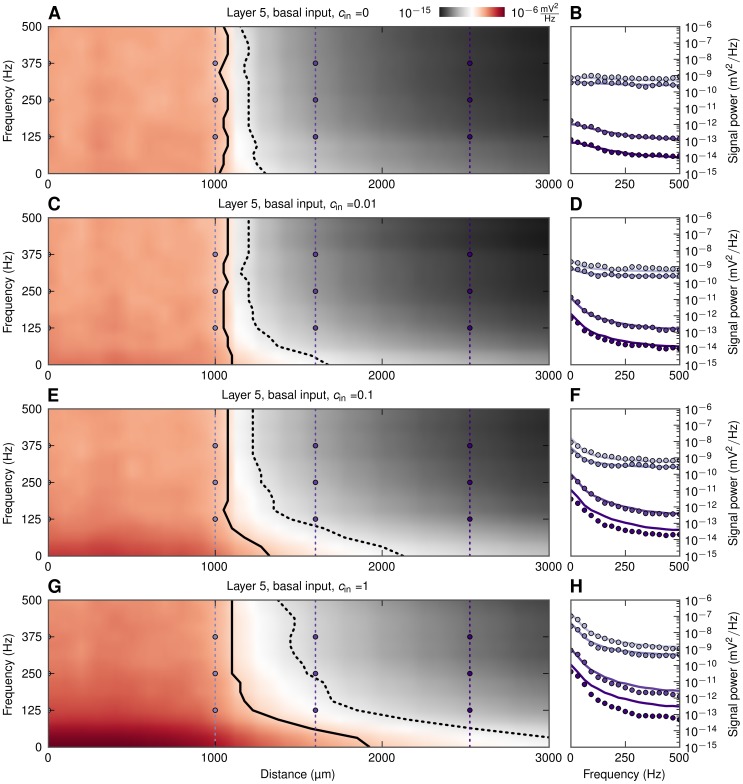
LFP signal power at the soma level as functions of frequency and distance from basally-activated pyramidal-cell populations. Colormaps (A, C, E, G) show the power of extracellular signal of a population of layer-5 cells receiving basal synaptic input for four levels of input correlation 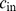 as functions of frequency and distance from center of populations. Black solid and dotted lines denote signal to noise ratio of 0.5 and 0.1, respectively. B, D, F, H: power spectra of extracellular signal at different distances, lines: prediction from simplified model in Equation 6, dots: full simulation. Thin vertical dotted lines with dots in A, C, E, G denote the distances at which the power spectra are shown, that is, at the center (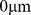), population edge (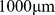), and two distances outside (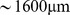 and 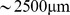).

We next investigated the related question of detectability, i.e., how far away from a synaptically activated population the generated LFP still can be detected above the ambient LFP ‘noise’. This noise level will naturally vary between experimental situations, but here we assumed it to be given by the background LFP signal from neurons of the same morphology, receiving the same number and type of synaptic inputs, except that the inputs are (1) uncorrelated and (2) homogeneously spread over the neuronal membrane. (The power of this background LFP signal is plotted as dotted lines in Figure 8.) The frequency-dependent signal decay and detectability outside basally-activated populations are illustrated in the 2D color plots in Figure 10. As in Figure 8, the population radius is fixed at 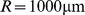, and we plot the PSD both inside and outside the population. The lines mark where the signal-to-noise ratio falls below 0.5 (solid line) and 0.1 (dotted line), respectively. Here the signal-to-noise ratio is defined as the ratio between the root mean square amplitudes of the LFP signal (from the basally-activated population) and the LFP noise (from the background population).

A first observation is that for uncorrelated synaptic inputs (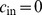, Figure 10A–10B), there is very little variation with frequency. Also the detectability of the LFP outside the active population is poor: the signal-to-noise ratio falls to 0.5 about 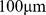 outside the population, and below 0.1 less than 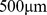 outside. The situation is seen to be very different when the populations receive correlated synaptic inputs. Focusing first on the case with the largest level of input correlations (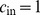, Figure 10G, 10H), we see that the lower frequencies of LFP extend further outside the population than the higher frequencies. For example, for the near-DC component (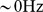) the signal-to-noise ratio is seen to be almost 0.5 at a distance of 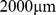, i.e., 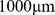 outside the population edge, and 0.1 as far way as 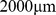 outside this edge. For the 125 Hz component, on the other hand, the signal-to-noise ratio is reduced to 0.5 as little as 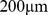 outside the population. The results for the intermediate cases (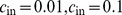) depicted in Figures 10C–10F are seen to bridge these uncorrelated and strongly correlated cases.

**Figure 11:**
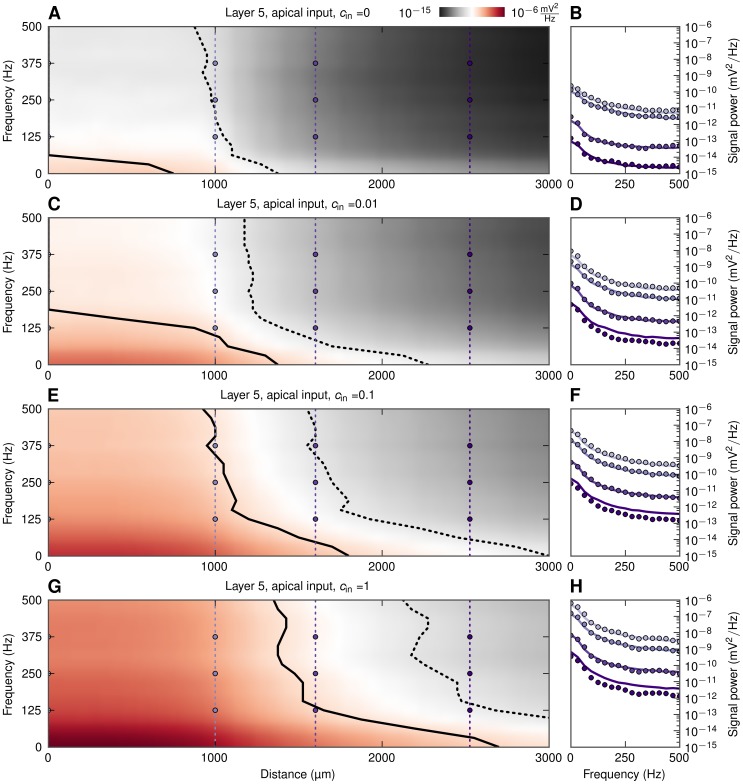
LFP signal power at the soma level as functions of frequency and distance from apically-activated pyramidal-cell populations. Colormaps (A, C, E, G) show the power of extracellular signal of a population of layer-5 cells receiving apical synaptic input for four levels of input correlation 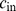 as functions of frequency and distance from center of populations. Black solid and dotted lines denote signal to noise ratio of 0.5 and 0.1, respectively. B, D, F, H: power spectra of extracellular signal at different distances, lines: prediction from simplified model in Equation 6, dots: full simulation. Thin vertical dotted lines with dots in A, C, E, G denote the distances at which the power spectra are shown, that is, at the center (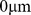), population edge (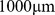), and two distances outside (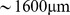 and 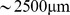).

The results for the basally-driven pyramidal cell population in Figure 10 demonstrate a main result from this study, namely that correlations in synaptic inputs may significantly enhance the amplitude and thus also the detectability of the low-frequency LFP components relative to the high-frequency LFP components. The same effect is observed for the same population when the synaptic inputs are placed solely on the apical part of the neurons, cf. Figure 11. However, here a sizable low-pass filtering effect in detectability is observed also for the case with uncorrelated input (Figure 11A, 11B) due to the intrinsic dendritic filtering effect [40], [42]. It is also worth noting that populations of layer-5 cells stimulated apically yielded the farthest-reaching LFP signal of all cases analyzed. Note also that the low-pass filtering effect in the boosting of LFP signal with increasing correlations was seen to be largely absent in the case of a spatially homogeneous distributions of synaptic inputs onto populations made of any of our three example neuronal morphologies (results not shown).

Finally, inspection of Figure 8 (and the PSD line plots in Figures 10 and 11) reveals that the predictions from the simplified model (Equation 6) agree excellently with the full numerical simulations for the case of uncorrelated input. However, the simplified model systematically overestimates the signal power for correlated populations for positions far outside the active populations. This is because the simplified model predicts a fall-off of the LFP amplitude proportional to 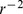 in the far-field limit, while in the full simulations the total LFP signal will be dominated by correlated dipoles oriented vertically. When moving horizontally from a a vertical dipole at a fixed vertical position, it follows from geometry that the dipole potential will decay as 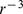 rather than 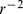 [40]. As a consequence the functional form of the lateral decay of the LFP signal outside a correlated population will be close to 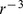 [34].

**Figure 12:**
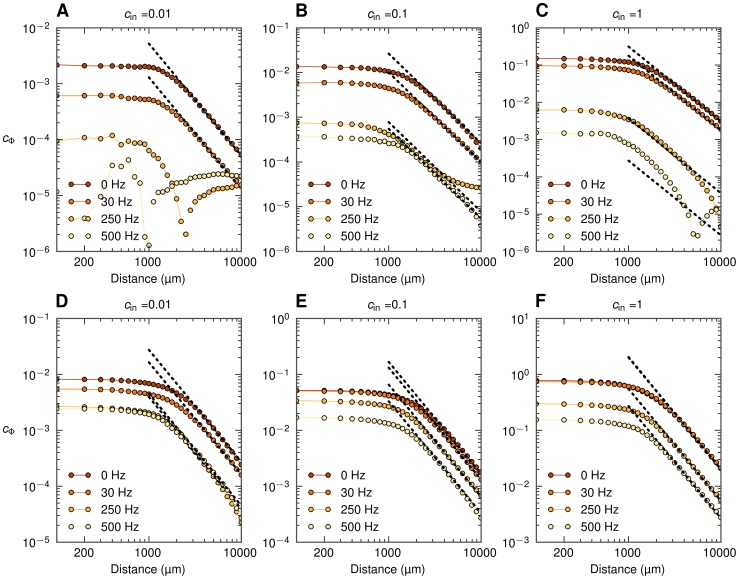
Population-averaged LFP coherence 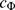 at the soma level as a function of distance 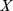 from center of population of layer-5 pyramidal cells. A, B, C: basal synaptic input, D, E, F: apical synaptic input. Dots not connected with lines indicate that 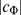 is plotted in place of spurious negative values (see Methods). Dashed lines mark 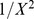 decay.

This limitation of the simplified model can be remedied by incorporating the fact that the evaluated population-averaged coherence 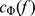 not only depends on the size of the population 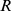 considered, but also on the electrode position 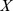 along the horizontal axis from where it is evaluated, i.e., 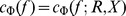. So far the population-averaged LFP coherence has been evaluated at the population center, i.e., at 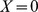. However, when Equation 17 is evaluated at other positions 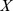, as shown in Figure 12, 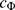 is observed to decay as 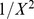 for 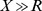. In the formula for the simplified model in Equation 6 the power 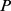 is in the correlation-dominated regime seen to be proportional to 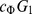. A modified simplified theory including not only the *X*-dependence of 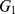 [34], [51], but also the observed 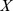-dependence of 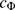 (i.e., 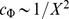 for 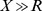), indeed predicts the correct far-field 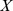-dependence outside the active population (see Figure S8). The physical interpretation is that the dominance of the LFP signal of the correlated vertical dipoles will be incorporated in the population-averaged LFP coherence 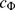.

### Depth dependence of LFP

**Figure 13:**
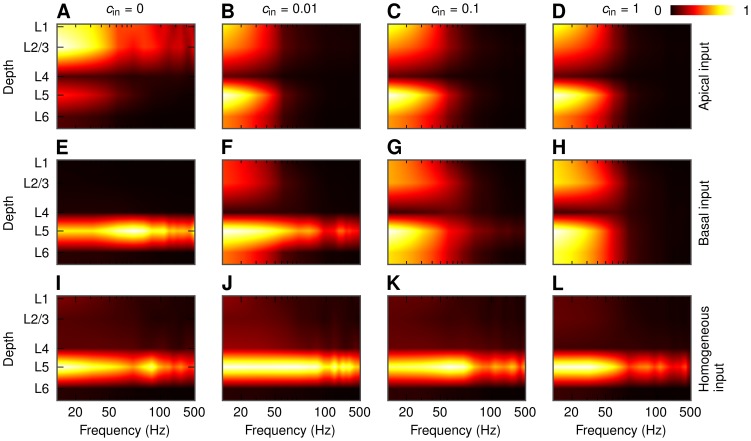
Depth-dependence of LFP power in the center of a population of layer-5 pyramidal cells. PSD of the LFP for different correlation levels and different patterns of synaptic input. Population radius: 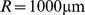. Values in each panel are normalized separately. A, B, C, D: apical synaptic input; E, F, G, H: basal synaptic input; I, J, K, L: homogeneous synaptic input. A, E, I: 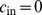; B, F, J: 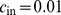; C, G, K: 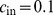; D, H, L: 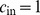.

Until now we have focused on the LFP calculated at the soma level of each population. However, in general there will be substantial transmembrane currents and thus LFP contributions across the entire dendritic structure [42]. Since the dendrites of the pyramidal cells span several cortical layers, it is natural to ask how the LFP power will depend on the depth. As for the soma-layer LFPs we observe in Figure 13 that the level of correlations is a crucial parameter also here. For example, for the case of uncorrelated (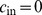), asymmetric synaptic inputs onto a layer-5 cell population the LFP is essentially located around the inputs (superficial layers in Figure 13A, layer 5 in Figure 13E). However, for strongly correlated synaptic input we instead obtain a dipolar, ‘dumbbell’ pattern with two poles in each end of the dendritic structure of the neuron (Figure 13B–D, Figure 13F–H). Similar behavior can be observed for the population of layer-3 pyramidal cells (Figure S9). The dipolar structure is not present in case of homogeneous synaptic input onto a layer-5 cell population (Figure 13I–L) and for a population of layer-4 cells (not shown). Figure 13B–D,G,H also reveals the same substantial boosting of the low-frequency (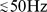) dumbbell-shaped LFPs for correlated synaptic inputs as previously seen in Figure 6. For symmetric or uncorrelated inputs, on the other hand, there is no such boosting, and less relative attenuation of the signal is observed at the higher frequencies.

**Figure 14:**
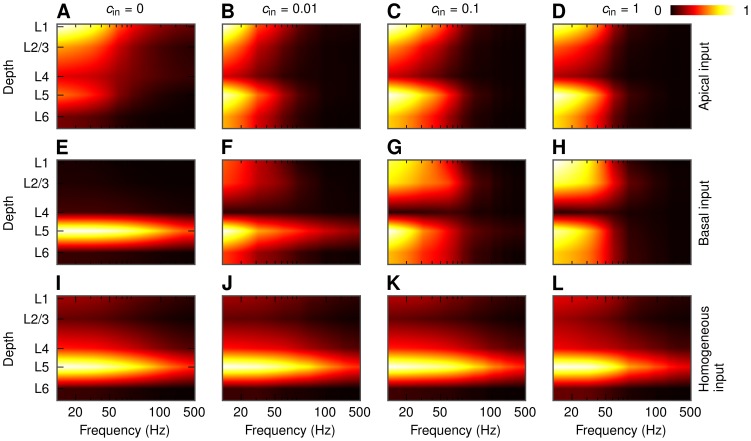
Simplified-model predictions of the depth-dependence of LFP power in a population of layer-5 pyramidal cells. PSD of the LFP for different correlation levels and different patterns of synaptic input as predicted by the simplified model of the population LFP. Population radius: 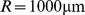. Values in each panel are normalized separately. A, B, C, D: apical synaptic input; E, F, G, H: basal synaptic input; I, J, K, L: homogeneous synaptic input. A, E, I: 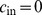; B, F, J: 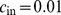; C, G, K: 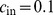; D, H, L: 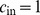.

**Figure 15:**
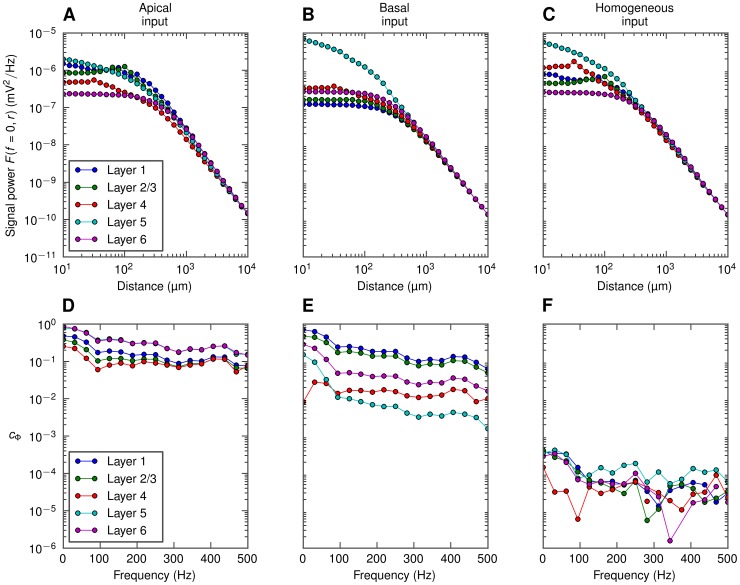
Ingredients of the simplified model of the depth-dependence of LFP power. Top row: squared shape functions 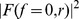 for the lowest-frequency component (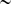
0 Hz) of the LFP generated by layer-5 cells with apical (A), basal (B) or homogeneous (C) synaptic input, at different recording depths. Bottom row: population-averaged LFP coherence 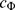, calculated at different depths in a maximally correlated (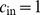) population of layer-5 cells with either apical (D), basal (E), or homogenous (F) distribution of synapses.

Interestingly and encouragingly the simplified model for the population LFP in Equation 6 captures, as seen in Figure 14, the salient features of the depth-dependence well. Now the shape curves 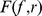 and the population-averaged coherence 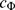 depend both on depth and lateral position, as well as frequency, as depicted in Figure 15. These functional dependencies of the elements of the simplified model also explain *why* the dumbbell LFP pattern arises for correlated, asymmetric synaptic inputs: As described in [34], [51] contributions from distant neurons (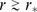) will dominate over neurons close by (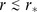) for correlated inputs, and as seen in Figure 15A–B for these distant neurons the shape functions 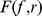 are not too different in magnitude in the various layers. As a consequence substantial LFPs (which more detailed analysis reveal to have a dumbbell structure) are thus seen at most cortical depths. For uncorrelated inputs (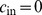), or homogeneously distributed correlated inputs resulting in very small correlations between the individual LFP contributions (Figure 15F), the neurons close by (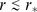) will dominate. Then for the case of basal input, for example, the somatic LFP (layer-5) will be much larger than the LFP in the other layers.

The dipolar LFP patterns observed for highly correlated synaptic input are consistent with the patterns observed in [9], where strongly correlated inputs was implicitly assumed in their more simplified scheme for calculating population LFPs (see Figure 13 therein).

## Discussion

In this computational study we have investigated the frequency dependence of the signal power and ‘locality’ of cortical local field potentials (LFP). While some low-pass filtering effects of the LFP are seen also for populations of cells receiving uncorrelated synaptic inputs or homogeneously distributed correlated synaptic inputs, the large frequency-dependent effects are seen when populations of pyramidal neurons receive correlated and spatially asymmetric inputs (i.e., either only basal or apical). For example, for the case with a layer-5 population receiving correlated, Poissonian synaptic currents (with a white-noise, i.e., flat band, power spectra) onto their basal dendrites, the power of the low-frequency LFP (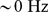) was seen to be an order of magnitude larger than the LFP power at 60 Hz. Correspondingly, the low-frequency LFP components were seen to extend much further outside the active population than high-frequency components.

The correlation of synaptic input currents and their spatial placement were observed to be equally crucial for determining the vertical profile of the LFP signal. For correlated and spatially asymmetric inputs, characteristic dipolar ‘dumbbell’ LFP structures spanning the vertical extent of the dendrites of the pyramidal neurons in the populations were observed; for uncorrelated and/or spatially homogeneous inputs, the LFP was instead confined around the positions of the somas (with the exception of uncorrelated apical input onto the layer-5 population).

The findings from our comprehensive biophysical simulations using reconstructed neuronal morphologies were backed up by a simplified model, adapted from [34], for generation of population LFP. This model is based on three factors: (1) the decay of the single-neuron contribution with the distance from the electrode represented by the frequency-dependent *shape function* 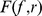, (2) the population geometry and density of neuronal LFP sources, and (3) the frequency-dependent *correlation* (or, more precisely, *coherence* 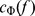) of the single-neuron LFP contributions from individual neural sources. Our simple model for the population LFP (Equation 6) was found to give quantitatively accurate predictions, implying that it captures the salient features. While some of the observed low-pass filtering could be traced back to single-neuron properties and the intrinsic dendritic filtering effect [40], [42] accounted for by the *shape function* 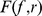, most of the observed low-pass filtering was due to strong low-pass filtering in the *coherence* 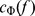 between the single-neuron LFP contributions: synaptic-input correlations translated into correlated single-neuron LFP contribution to a much larger extent for lower frequencies than for higher frequencies. As a direct consequence, the low-frequency components of the extracellular potential are significantly boosted in populations with correlated synaptic input. In our model this happens purely because of dendritic filtering, as the synaptic input currents themselves have been tailored to have a flat (white-noise) PSD. With a colored (frequency-dependent) spectrum of the synaptic input, the power spectrum of the LFP would be given as the product of the PSD of this synaptic filter and the PSD from the dendritic filtering investigated here (cf. Figure 2D and 2E).

A key qualitative finding in our study is that the size of the signal-generating region, i.e., the *spatial reach*, may in the case of correlated synaptic input vary strongly with frequency. For the example population in Figure 1 we see that for 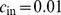, a plausible correlation level in cortical spiking networks (see, e.g., Figure 6 in [34]), the LFP spatial reach may be reduced from close to the size of the population (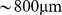) for 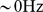 to 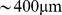 for 60 Hz. For uncorrelated input, however, the spatial reach will generally always be small (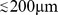) for all frequencies, with the exception of the case with apical input on large pyramidal cells (Figure 7). Note that in the present simulation scheme the spatial reach is by definition less than 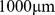, the size of our model population. Unlike for uncorrelated populations, the LFP power will for correlated populations keep on increasing when the population grows beyond 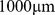 [34]. The present definition of spatial reach (95% of the amplitude for 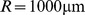) thus underestimates the true size of the signal-generating region in this case.

In a recent experimental study from macaque auditory cortex [33] it was observed that different frequency bands spread equally far from a source (cf. Figures 5 and 6 there). There are, however, notable differences between this study and our present approach, making it difficult to compare the results. First, here we focus mostly on the spread of the LFP along cortical layers at the soma level, while in [33] the spread in vertical direction was studied. Second, and likely more importantly, in [33] the LFP amplitude at a given latency after stimulation was used to extract LFP decay profiles. In contrast, we here use noise input and consider the root mean square amplitude of LFP over a relatively long time period. Further, the correlation level of the synaptic input, found here to be a critical parameter in determining the frequency dependence, is not known in the situation in [33]. It is thus difficult to assess whether our results are in accordance, or not.

Our results have direct consequences for the interpretation of observed cross-correlations between extracellular potentials recorded at different electrodes [52]–[57]. As demonstrated here the low-frequency LFP signal generated by a population of neurons around one electrode receiving asymmetric synaptic input, may extend a millimeter or more outside the active population (see, e.g., Figure 10G). Thus measured correlations in the low-frequency LFP components between two electrodes positioned, say, one millimeter apart, may be due to volume conduction effects. However, cross-correlation induced by such volume conduction will, as demonstrated here, have a diminishing spatial range with increasing LFP frequencies. Note also that the magnitudes of the LFP amplitude at the two adjacent electrodes will aid in the interpretation: while volume conduction may propagate the LFP a millimeter or more, the amplitude will rapidly diminish with distance (cf. Figure 10 and 11). Thus the observation of large-amplitude LFPs at both electrodes would be an indication that both electrodes are surrounded by strong LFP-generating populations.

In [58] the temporal power spectra of the EEG were shown to be well fitted by 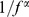 power-law functions with power-law exponents 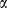 varying between brain areas: in the frontal lobe 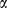 was reported to be 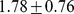, while in the occipital lobe 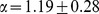. Power laws have also been found in recordings of the LFP, see, e.g., [59], [60], often with different exponents 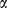. In [60] 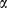 was shown to vary between network states, more specifically between the slow-wave sleep and awake states. In this context it is interesting to note that the PSDs in our Figure 3F express approximate power laws with exponents 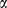 highly dependent on the degree of coherence. This finding suggests that varying levels of coherence in the synaptic input may be a mechanism underlying the different experimentally observed power laws. This would also be in agreement with the experimental observations that network states with a presumably large coherence (e.g., slow wave sleep in [60]) typically express a larger value of 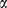 than network states for which the coherence is lower (e.g., awake state in [60]).

In our modeling we have assumed the extracellular medium to have a frequency-independent conductivity, an assumption supported by a recent thorough experimental study of the electrical properties of monkey cortical tissue [61]. However, if for example low-frequency filtering 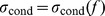 of the extracellular medium should be found [62], this filtering would superimpose directly on the filtering seen here, i.e., the total LFP filter would be the product of the LFP filter calculated here and the filter from the extracellular medium (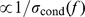).

Here we have focused on the spatial and spectral properties of LFP signals triggered by presynaptic spikes that could originate from within the same cortical population or come from other distant brain regions. While not addressed here, it may be that the LFP signal itself influences the timing of these locally generated spikes through ephaptic coupling [63], [64]. That would in turn influence the correlation structure of incoming spikes and thereby also the generated LFP signal. Since our simulations show that both the LFP amplitude and spatial reach is larger for low than for high frequencies, this suggests that if ephaptic effects play a role in cortical processing, they would likely be larger for low than for high frequencies.

The present study has focused on LFPs generated by synaptic input currents and the associated return currents. While these synaptic contributions are thought to dominate at least low-frequency LFPs [9], [41], [65], other sources will also contribute to the signal in the frequency band typically associated with the LFP (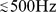). Sodium spikes, i.e., the fast extracellular signatures of action potentials, may contribute to the LFP signal for frequencies as low as 100 Hz [40], [44]–[47], and slower phenomena such as calcium spikes and spike afterhyperpolarization [66] at lower frequencies still. For spikes the source of the LFP is active sodium and potassium conductances localized mainly in the soma and axon hillock, rather than synaptic currents that can be positioned all over the dendrites. Nevertheless, many of our present observations and findings also apply here, in particular, the intrinsic dendritic filtering effect that will give faster decay with distance of the single-neuron contributions for high frequencies than for low frequencies [40] and the possibility of amplification of the population signal when neuron spiking is highly correlated. Interestingly, the latter effect has recently been demonstrated in a very accomplished biophysical modeling study to be the likely mechanism behind the large LFP power observed in the 100–200 Hz frequency band in rat hippocampus [44].

In the present analysis we have modeled the dendrites as simple RC-circuits which, in combination with the use of current-based synapses, made the system linear. This greatly facilitated the present frequency-resolved analysis in that the LFPs at different frequencies were effectively decoupled, cf. the standard theory for Fourier analysis of linear systems. The present results also serve as a starting point for the exploration of non-linear effects, for example due to active membrane conductances. Close to the resting potential of the neuron, the active conductances can be linearized, and the neuron dynamics can be described by linear theory with quasi-active membrane modeled by a combination of resistors, capacitors and inductors (see, e.g., Ch. 10 in [67], Ch. 9 in [68], or [69]). At present it is not known to what extent such ‘generalized’ linear schemes will be able to account for the LFP generation in real neurons, but the present forward-modeling scheme, applicable for passive and active conductances alike, can be used to explore this question systematically.

## Methods

### LFP simulations

The setup of the LFP simulations is almost identical to the scheme used to model cortical population LFPs in [34]. The main difference is that here we use a much smaller synaptic time constant to achieve an effectively white (flat) power spectrum for the synaptic currents for the frequencies of interest here (less than 500 Hz). We therefore also use a smaller numerical time step. The model parameters are presented in detail (in the format described in [70]) in Tables S1, S2 and S3. For the reader's convenience we summarize the essential information below.

#### Cell models

We analyze three compartmental cell models: the layer-3 and layer-5 pyramidal cells, and layer-4 stellate cells [71], available from ModelDB [72], accession number 2488. We modified the models by removing active conductances and axon segments. The passive parameters of the cells were the following: specific axial resistance 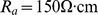, specific membrane resistance 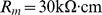, specific membrane capacitance 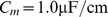.

Each simulated cell was stimulated using 1000 excitatory current-based 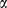-function synapses with a time constant 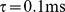. The synaptic time constant was short enough to ensure that the spectrum of the input current was flat in the studied range. Each synapse was driven by a homogeneous Poisson spike train with the rate of 5 spikes per second. The spike trains driving one cell were independent. For uncorrelated input into the population also the spike trains belonging to each cell were independent, for correlated input they were drawn (without repetitions for each cell) from a common pool consisting of 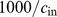 spike trains. As a result, in case of correlated input each two cells shared 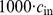 spike trains on average. Note that even for 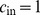, when each of the cells is driven by the same spike trains, the spike trains will in general be assigned to different synaptic locations.

We simulated activity of cells for either 10200 ms (single-cell shape functions at the soma level and LFP in the population's center at the soma level) or 1200 ms (LFP at points not in the population's center and and LFP shown in Section on depth dependence of LFP). The first 200 ms were discarded to avoid start-up artifacts. We used a fixed time step of 1/64 ms, and recorded the results of the simulation (transmembrane currents in all compartments) with 1 ms time step (sampling frequency 1 kHz).

For the pyramidal cells we employed three stimulation patterns: the synapses were distributed either in the apical or basal part, or homogeneously throughout the whole dendritic tree (in each case the probability of attaching a synapse in a given compartment was proportional to its surface area). We used the same layer boundaries and soma depths as in [34].

#### Calculation of LFP

The extracellular electric potential was calculated using the line-source method [37], [73], resulting from integration of Equation 1 over linear dendritic segments. We assumed a purely resistive, homogeneous, isotropic and infinite extracellular medium, and an ideal point electrode (no filtering), placed either at the soma level (single-depth simulations), or at the middle depth of each layer (simulations of depth-dependence of the LFP). In single-cell simulations the electrode was placed at a distance (between 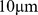 and 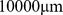) from a single cell, in population simulations it was placed either at the center of the population or at 31 points placed between 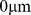 and 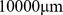 from the center. To obtain the model LFP at the center of differently-sized populations we summed contributions from different subsets (cells located closer to the electrode than some distance) of the same full (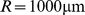) population.

#### Single-cell shape functions

To obtain single-cell shape functions (Figure 3A) we calculated the LFP at different distances from a single cell, then calculated power spectra of these signals. The final curves were obtained by averaging power spectra from 100 simulations for each distance.

#### Population simulations

We simulated populations of 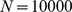 identical neuron. The cells were placed homogeneously within a disc of 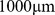 radius at the same depth. Each cell was rotated randomly along the vertical axis.

#### Software

We performed the simulations using the NEURON simulator (www.neuron.yale.edu, [49]) and the Python (www.python.org) interface to NEURON [74]; we also used NeuroTools (neuralensemble.org/trac/NeuroTools/). The calculations of extracellular field were performed using LFPy [75] — Python package for modeling of LFP.

### Derivation of the mean-field model

To derive the formula in Equation 6 for the power spectral density (PSD) of the extracellular signal in the center of the population we start with the assumption that 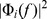, the PSD of the contribution of the *i*-th cell at given frequency 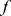, may be factorized as
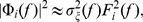
(8)where 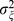 is the PSD of the input current, and 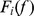 is the frequency-dependent *shape function* of the *i*-th cell. We also assume that the shape function 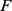 depends only on frequency and distance from the center, that is:
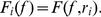
(9)


Let us compute the PSD of the population signal 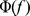 (dependence on frequency 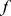 dropped below for convenience):
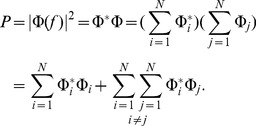
(10)We now use Equations 8 and 9 to express 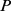 in terms of shape functions and the PSD of the input current, note the trick (multiplication by 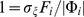) in the double sum: 
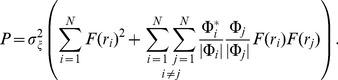
(11)We further assume that the coherence term 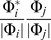 may by replaced by its population average over 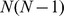 pairs. This assumption, while not true in general, is a reasonable approximation because the input correlations are homogeneous across the population. We can then move the coherence term in front of the double sum:
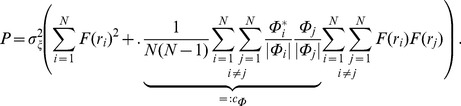
(12)As marked in Equation 12, we denote the population-averaged coherence by 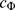. We further rewrite 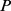 as
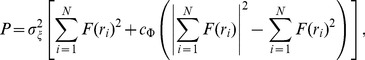
(13)and finally
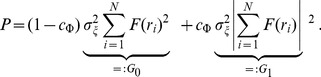
(14)


If we assume approximate, power-law shape functions 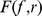 parametrized by the cutoff distance 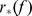 (Equation 7), and change sums to integrals as in Equations 4 and 5 (limit of large number of cells), then the functions 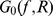 and 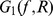 have the following closed-form representation [51]:
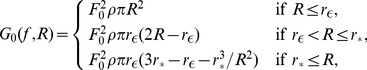
(15)

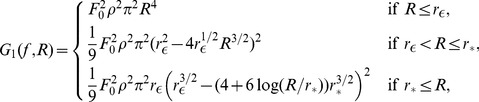
(16)which we used for calculating predictions from the simplified model. At the soma level we effectively set 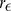 to zero; for modeling the LFP at any different layer we used 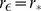 [51]. The model can be modified to calculate the power of the signal outside the center of the population, i.e., at positions offset from the center by the distance 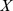. In that case, the function 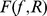 in (4) and (5) has to be replaced by 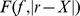. It is no longer easy to obtain closed-form formulae for 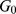 and 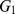 in terms of 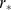, and we used the (non-parametric) shape curves obtained from the simulations, as the final integration had to be done numerically anyway.

### Data analysis

#### Population-averaged LFP coherence

It is hard to estimate the population-averaged LFP coherence 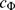 directly as an average of pairwise coherences between the single-cell contributions to the LFP. Therefore, we used the same technique as in [34] (Equations 14 and 15 therein), ending up with
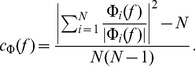
(17)Coherence is always positive; however, the population-averaged coherence 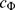 estimated using Equation 17 may take spurious negative value (for example because finite-length signals are used). This does not mean that 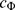 is truly negative, but rather that the value is too small to be estimated reliably from the amount of data available. In such cases we plotted 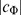 in figures.

Note that 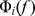 in Equation 17 may be evaluated either at the population center, or at a lateral position 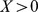; as a result we will get either 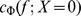 or 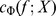, see Section on lateral decay of LFP outside the population.

#### Frequency analysis

To calculate the power spectral densities we used the Welch's average periodogram method (the matplotlib.mlab.psd function from Matplotlib [76]). We used a Hanning window of length 32 or 128 time steps (32 or 128 ms) and overlap between blocks equal to the half of the window length, which resulted in 17 (or 65) equally spaced frequency bins between 0 and 500 Hz. When calculating the population-averaged LFP coherence, Equation 17, we calculated the discrete Fourier transform and binned the resulting 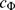 in the same frequency bins as resulting from the Welch's average periodogram method.

#### Spatial reach of LFP

To obtain the spatial reach of the LFP we calculated the power spectral density 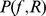 of the population LFP as a function of frequency 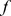 and population radius 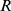 (taking values between 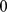 and 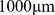 in 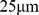 increments). The spatial reach at given frequency was defined as the smallest radius 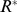 for which the amplitude 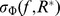 is larger than 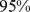 of the amplitude calculated for the full population.

#### Software

Data analysis was performed using NumPy and SciPy Python packages [77] and IPython [78]. Plotting was done using Matplotlib [76].

## Supporting Information

Figure S1
**Power spectral density of population LFP as a function of frequency and the population radius.** Full simulation results (dots) and simplified model predictions (lines) for the soma-level LFP at the center of disc-like populations of layer-5 pyramidal cells receiving basal synaptic input. Three different input correlation levels 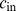 are considered. A, B, C: PSD of population LFP for three population radii 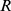. D, E, F: dependence of power of three different frequency components on the population radius 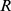. This is an alternate version of Figure 6 from the paper; here the coherence 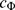 is estimated not just once for the full (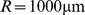) population, but in a radius-dependent fashion, for each population radius 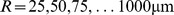 separately. In effect the simplified model predictions are closer to the full simulations than in Figure 6.(TIFF)

Figure S2
**The shape function** 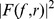 **and the population LFP power spectra at the soma level for layer-5 cells with apical input.** A. Spatial decay in lateral direction for the squared single-cell shape functions 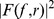 for three different frequencies f = 0, 60 and 500 Hz. B. Single-cell LFP spectra 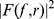 for three different lateral distances from the soma (dotted vertical lines in A). C. Power spectra 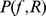 of the compound LFP (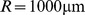); dots correspond to simulation; lines correspond to predictions from the simplified model.(TIFF)

Figure S3
**The shape function** 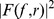 **and the population LFP power spectra at the soma level for layer-5 cells with homogeneous input.** A. Spatial decay in lateral direction for the squared single-cell shape functions 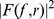 for three different frequencies f = 0, 60 and 500 Hz. B. Single-cell LFP spectra 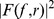 for three different lateral distances from the soma (dotted vertical lines in A). C. Power spectra 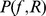 of the compound LFP (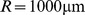); dots correspond to simulation; lines correspond to predictions from the simplified model.(TIFF)

Figure S4
**The shape function** 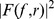 **and the population LFP power spectra at the soma level for layer-3 cells with apical input.** A. Spatial decay in lateral direction for the squared single-cell shape functions 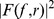 for three different frequencies f = 0, 60 and 500 Hz. B. Single-cell LFP spectra 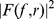 for three different lateral distances from the soma (dotted vertical lines in A). C. Power spectra 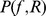 of the compound LFP (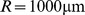); dots correspond to simulation; lines correspond to predictions from the simplified model.(TIFF)

Figure S5
**The shape function** 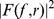 **and the population LFP power spectra at the soma level for layer-3 cells with basal input.** A. Spatial decay in lateral direction for the squared single-cell shape functions 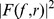 for three different frequencies f = 0, 60 and 500 Hz. B. Single-cell LFP spectra 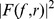 for three different lateral distances from the soma (dotted vertical lines in A). C. Power spectra 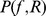 of the compound LFP (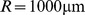); dots correspond to simulation; lines correspond to predictions from the simplified model.(TIFF)

Figure S6
**The shape function** 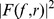 **and the population LFP power spectra at the soma level for layer-3 cells with homogeneous input.** A. Spatial decay in lateral direction for the squared single-cell shape functions 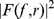 for three different frequencies f = 0, 60 and 500 Hz. B. Single-cell LFP spectra 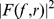 for three different lateral distances from the soma (dotted vertical lines in A). C. Power spectra 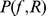 of the compound LFP (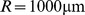); dots correspond to simulation; lines correspond to predictions from the simplified model.(TIFF)

Figure S7
**The shape function** 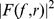 **and the population LFP power spectra at the soma level for layer-4 cells with homogeneous input.** A. Spatial decay in lateral direction for the squared single-cell shape functions 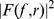 for three different frequencies f = 0, 60 and 500 Hz. B. Single-cell LFP spectra 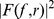 for three different lateral distances from the soma (dotted vertical lines in A). C. Power spectra 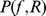 of the compound LFP (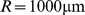); dots correspond to simulation; lines correspond to predictions from the simplified model.(TIFF)

Figure S8
**Decay of extracellular potential at the soma level outside populations of layer-5 cells with asymmetric input.** Each of the panels shows full simulation results (dots) and predictions from simplified model Equation 5 (lines) for one frequency band (0, 60, 500 Hz) and four input correlation levels. Horizontal dotted lines indicate ‘noise level’ (power of the signal generated by a population of uncorrelated cells with homogeneous input, see text). A, B, C: basal synaptic input. D, E, F: apical synaptic input. This is an alternate version of Figure 8 from the paper, here the population-averaged coherence 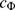 depends also on the lateral position of the electrode. In effect the simplified model predictions are closer to the full simulations than in Figure 8.(TIFF)

Figure S9
**Depth-dependence of LFP power in the center of a population of layer-3 pyramidal cells.** PSD of the LFP for different correlation levels and different patterns of synaptic input. Population radius: 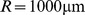. Values in each panel are normalized separately. A, B, C, D: apical synaptic input; E, F, G, H: basal synaptic input; I, J, K, L: homogeneous synaptic input. A, E, I: 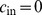; B, F, J: 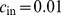; C, G, K: 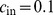; D, H, L: 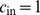.(TIFF)

Table S1 **Summary of the population model used for LFP simulations.** Continues in Table S2.
(PDF)

Table S2 **Summary of the population model used for LFP simulations.** Continued from Table S1.
(PDF)

Table S3 **Parameters of the population model used for LFP simulations.**
(PDF)
