# Supplementary figures and images for: Frequency Dependence of Signal Power and Spatial Reach of the Local Field Potential
Source: PLoS Comput Biol. 2013 Jul 18;9(7):e1003137. doi: 10.1371/journal.pcbi.1003137 (PMC3715549; doi:10.1371/journal.pcbi.1003137)

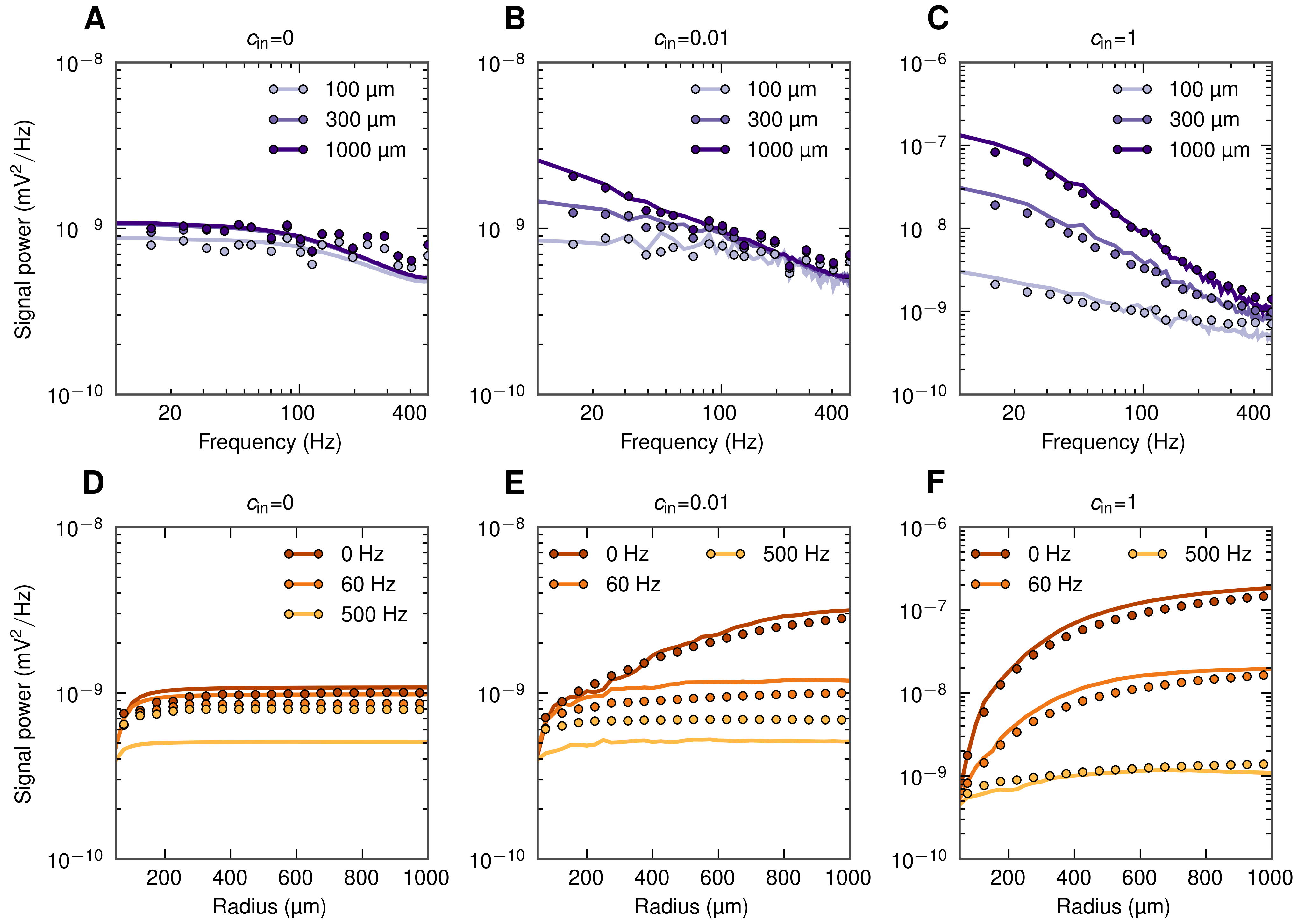

Supplement: Figure S1 — Power spectral density of population LFP as a function of frequency and the population radius. Full simulation results (dots) and simplified model predictions (lines) for the soma-level LFP at the center of disc-like populations of layer-5 pyramidal cells receiving basal synaptic input. Three different input correlation levels are considered. A, B, C: PSD of population LFP for three population radii . D, E, F: dependence of power of three different frequency components on the population radius . This is an alternate version of Figure 6 from the paper; here the coherence is estimated not just once for the full () population, but in a radius-dependent fashion, for each population radius separately. In effect the simplified model predictions are closer to the full simulations than in Figure 6. (TIFF) [file pcbi.1003137.s001.tiff]

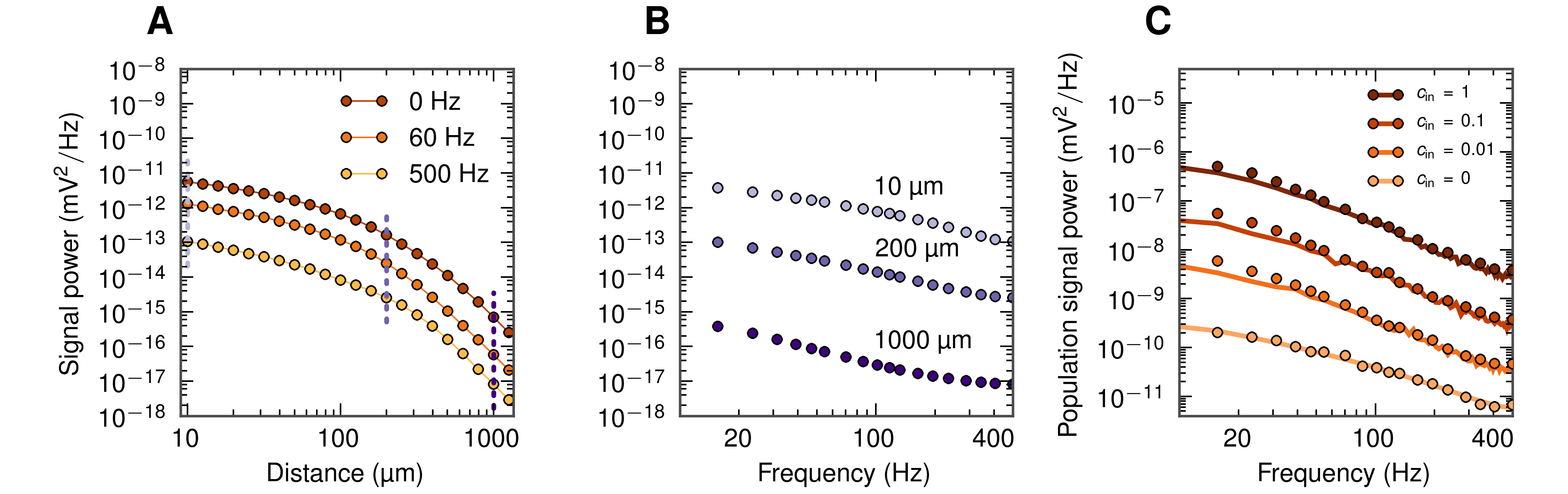

Supplement: Figure S2 — The shape function and the population LFP power spectra at the soma level for layer-5 cells with apical input. A. Spatial decay in lateral direction for the squared single-cell shape functions for three different frequencies f = 0, 60 and 500 Hz. B. Single-cell LFP spectra for three different lateral distances from the soma (dotted vertical lines in A). C. Power spectra of the compound LFP (); dots correspond to simulation; lines correspond to predictions from the simplified model. (TIFF) [file pcbi.1003137.s002.tiff]

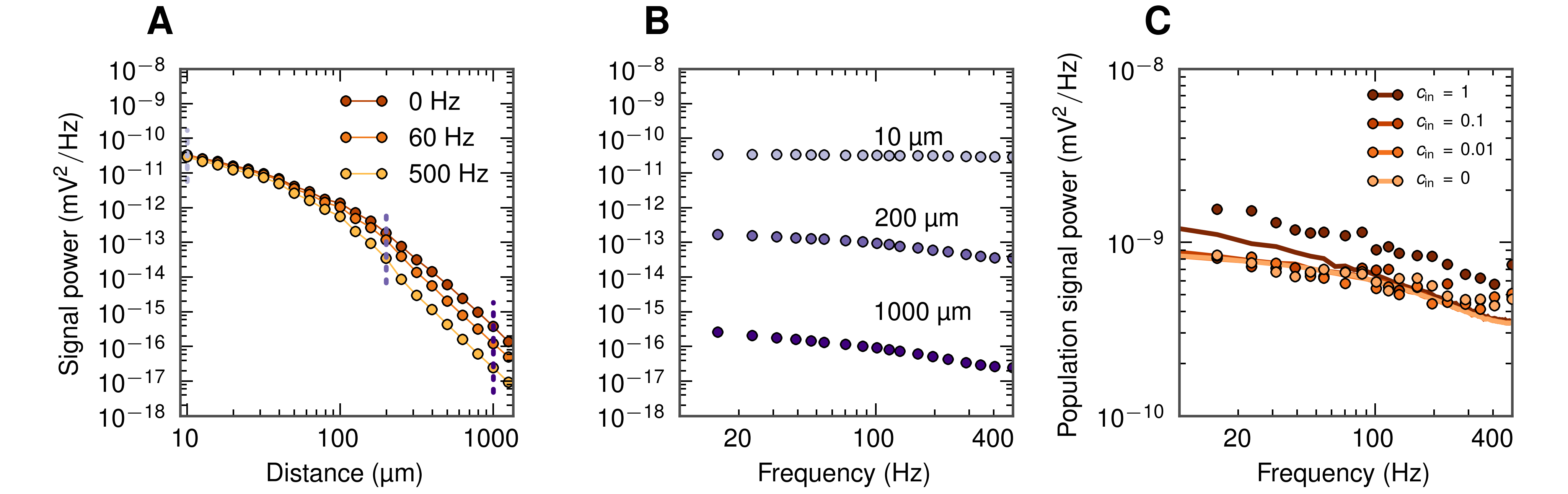

Supplement: Figure S3 — The shape function and the population LFP power spectra at the soma level for layer-5 cells with homogeneous input. A. Spatial decay in lateral direction for the squared single-cell shape functions for three different frequencies f = 0, 60 and 500 Hz. B. Single-cell LFP spectra for three different lateral distances from the soma (dotted vertical lines in A). C. Power spectra of the compound LFP (); dots correspond to simulation; lines correspond to predictions from the simplified model. (TIFF) [file pcbi.1003137.s003.tiff]

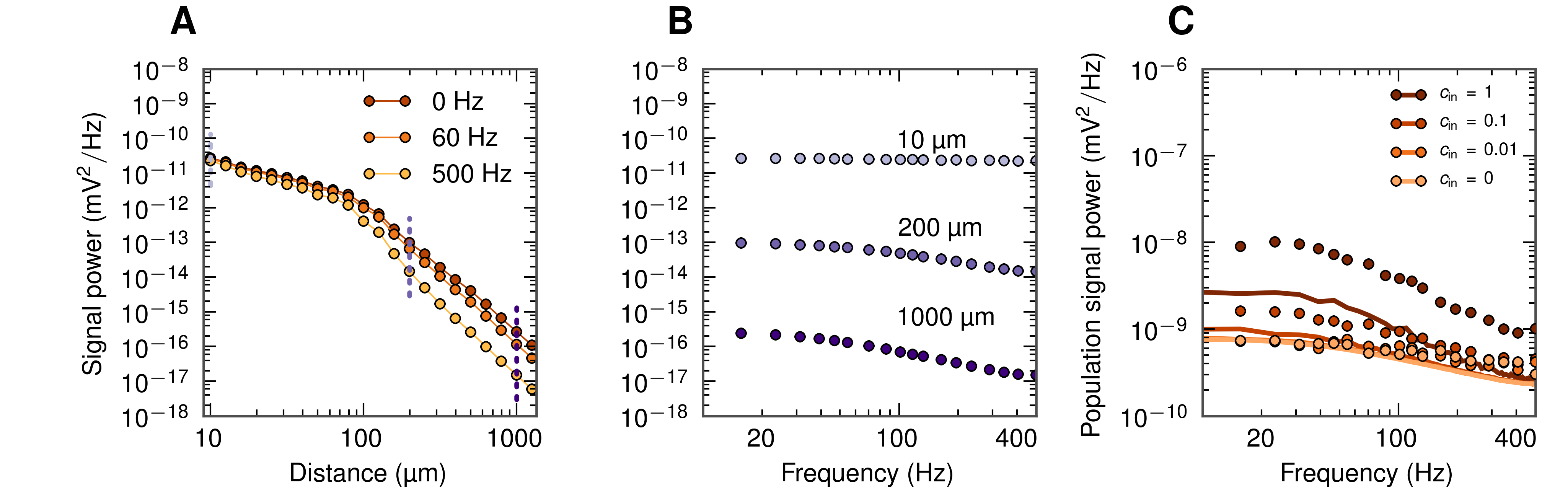

Supplement: Figure S4 — The shape function and the population LFP power spectra at the soma level for layer-3 cells with apical input. A. Spatial decay in lateral direction for the squared single-cell shape functions for three different frequencies f = 0, 60 and 500 Hz. B. Single-cell LFP spectra for three different lateral distances from the soma (dotted vertical lines in A). C. Power spectra of the compound LFP (); dots correspond to simulation; lines correspond to predictions from the simplified model. (TIFF) [file pcbi.1003137.s004.tiff]

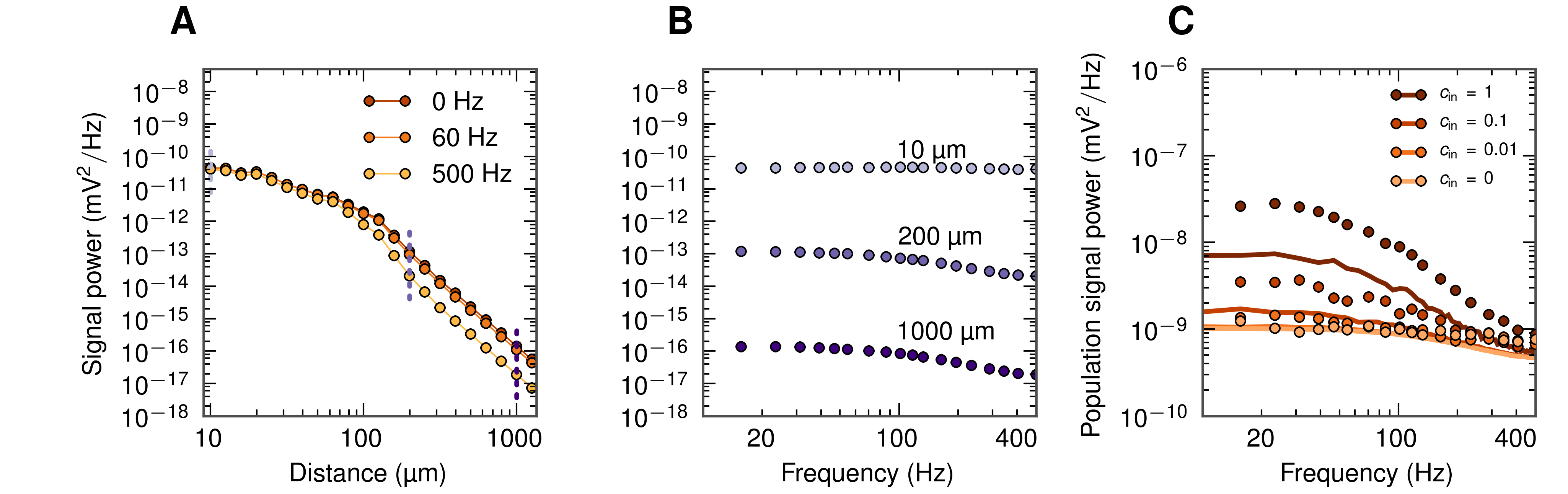

Supplement: Figure S5 — The shape function and the population LFP power spectra at the soma level for layer-3 cells with basal input. A. Spatial decay in lateral direction for the squared single-cell shape functions for three different frequencies f = 0, 60 and 500 Hz. B. Single-cell LFP spectra for three different lateral distances from the soma (dotted vertical lines in A). C. Power spectra of the compound LFP (); dots correspond to simulation; lines correspond to predictions from the simplified model. (TIFF) [file pcbi.1003137.s005.tiff]

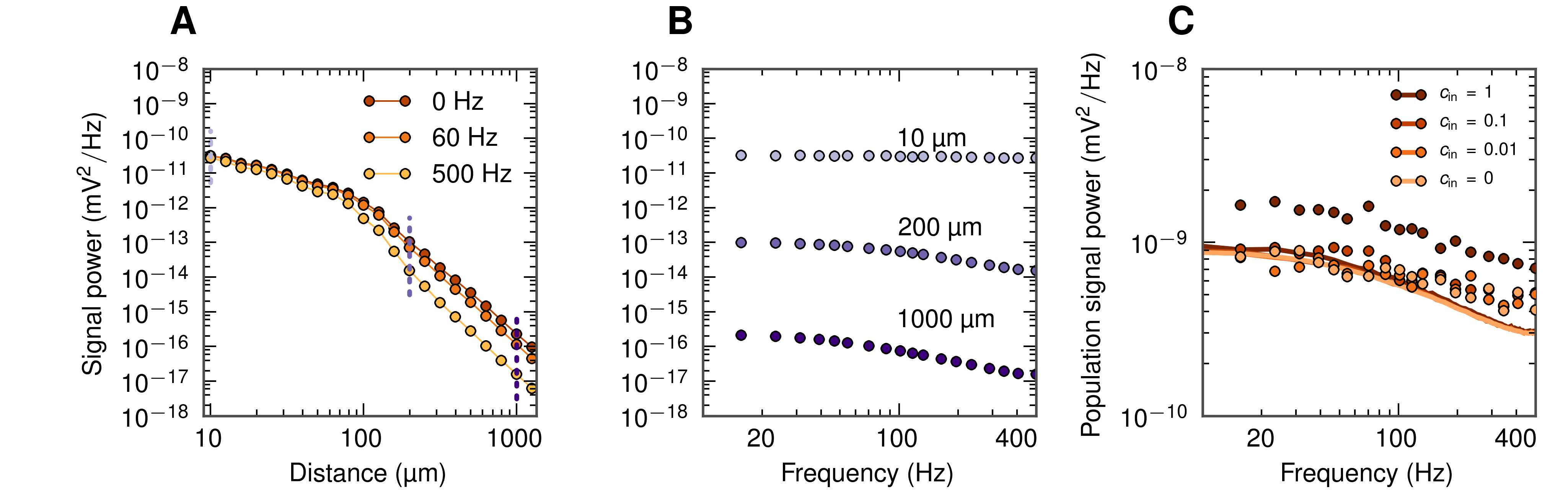

Supplement: Figure S6 — The shape function and the population LFP power spectra at the soma level for layer-3 cells with homogeneous input. A. Spatial decay in lateral direction for the squared single-cell shape functions for three different frequencies f = 0, 60 and 500 Hz. B. Single-cell LFP spectra for three different lateral distances from the soma (dotted vertical lines in A). C. Power spectra of the compound LFP (); dots correspond to simulation; lines correspond to predictions from the simplified model. (TIFF) [file pcbi.1003137.s006.tiff]

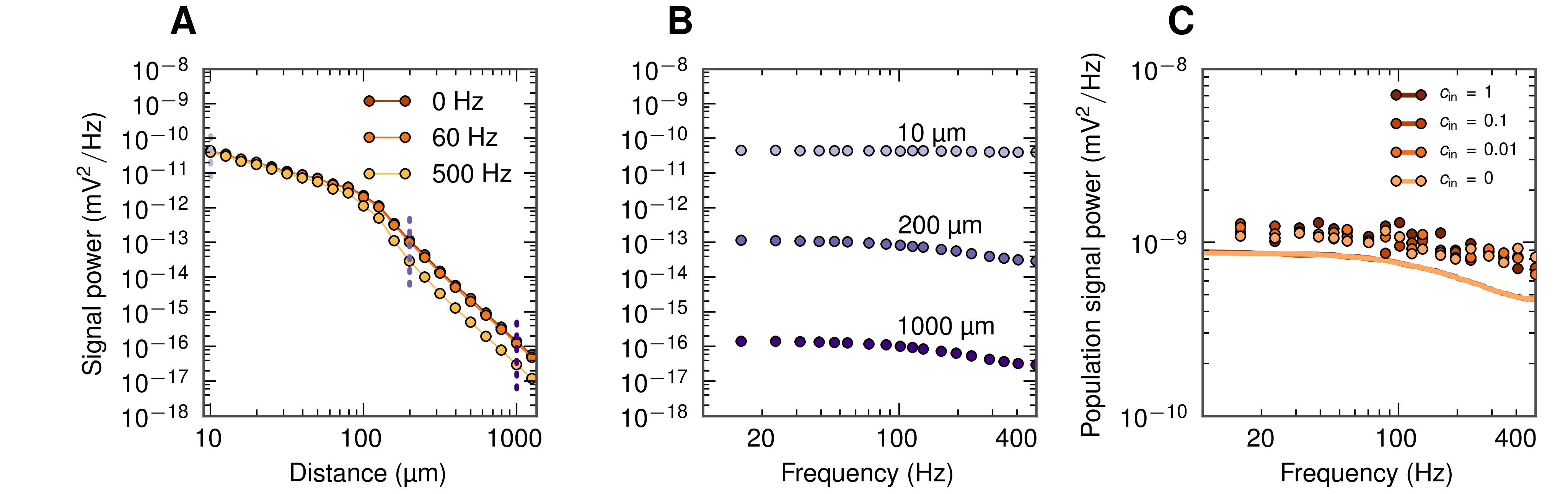

Supplement: Figure S7 — The shape function and the population LFP power spectra at the soma level for layer-4 cells with homogeneous input. A. Spatial decay in lateral direction for the squared single-cell shape functions for three different frequencies f = 0, 60 and 500 Hz. B. Single-cell LFP spectra for three different lateral distances from the soma (dotted vertical lines in A). C. Power spectra of the compound LFP (); dots correspond to simulation; lines correspond to predictions from the simplified model. (TIFF) [file pcbi.1003137.s007.tiff]

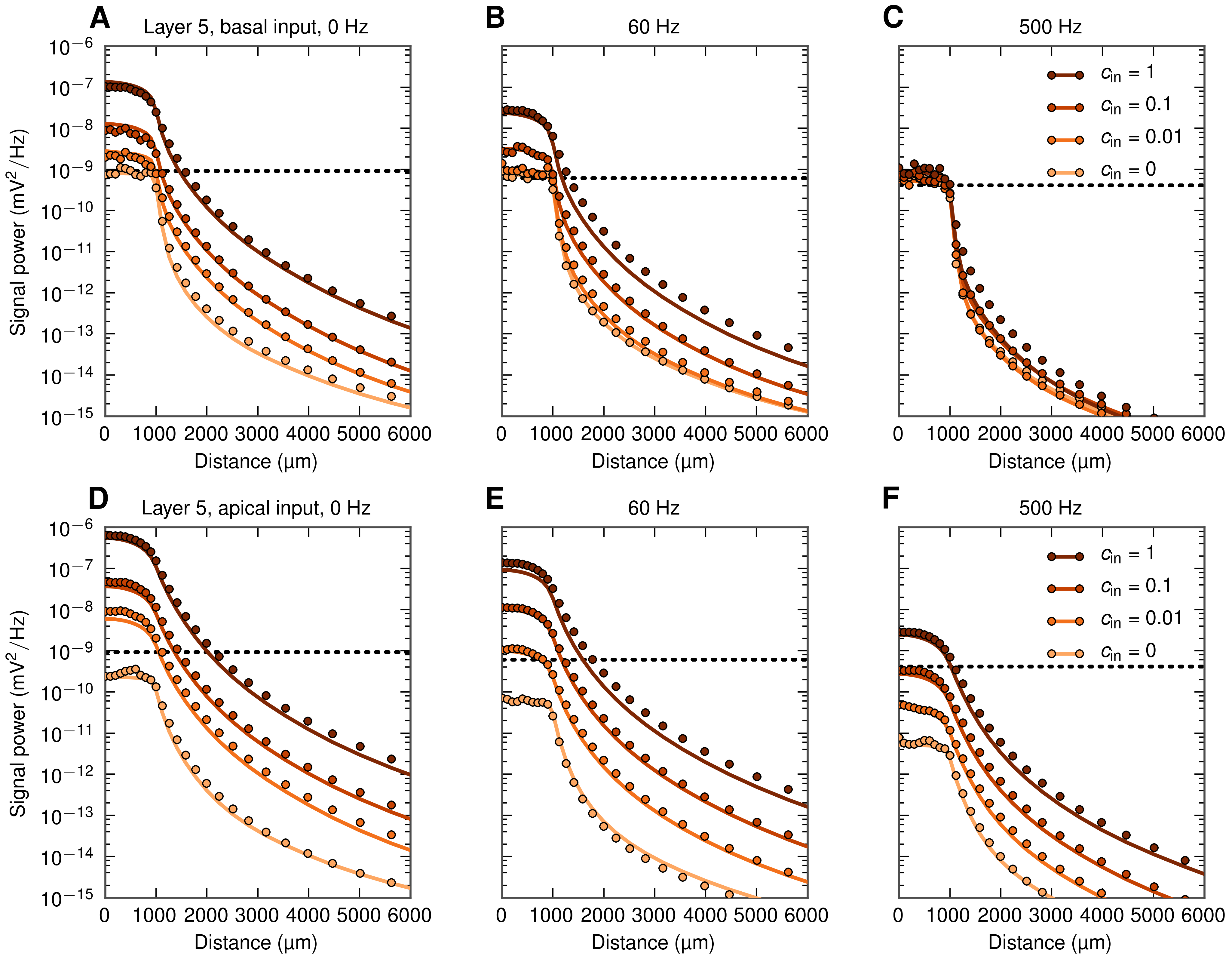

Supplement: Figure S8 — Decay of extracellular potential at the soma level outside populations of layer-5 cells with asymmetric input. Each of the panels shows full simulation results (dots) and predictions from simplified model Equation 5 (lines) for one frequency band (0, 60, 500 Hz) and four input correlation levels. Horizontal dotted lines indicate ‘noise level’ (power of the signal generated by a population of uncorrelated cells with homogeneous input, see text). A, B, C: basal synaptic input. D, E, F: apical synaptic input. This is an alternate version of Figure 8 from the paper, here the population-averaged coherence depends also on the lateral position of the electrode. In effect the simplified model predictions are closer to the full simulations than in Figure 8. (TIFF) [file pcbi.1003137.s008.tiff]

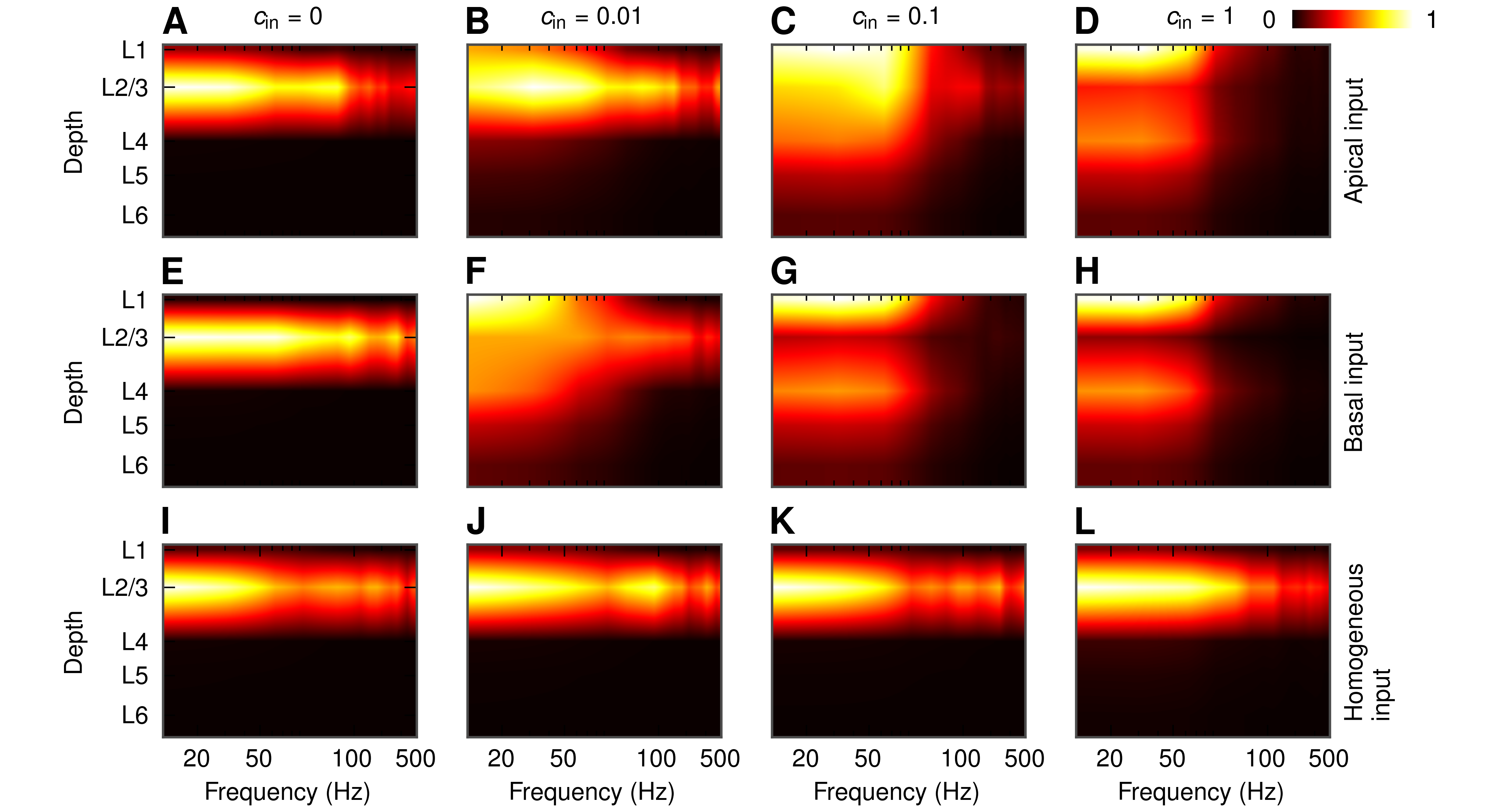

Supplement: Figure S9 — Depth-dependence of LFP power in the center of a population of layer-3 pyramidal cells. PSD of the LFP for different correlation levels and different patterns of synaptic input. Population radius: . Values in each panel are normalized separately. A, B, C, D: apical synaptic input; E, F, G, H: basal synaptic input; I, J, K, L: homogeneous synaptic input. A, E, I: ; B, F, J: ; C, G, K: ; D, H, L: . (TIFF) [file pcbi.1003137.s009.tiff]
