# Supplementary material for: Frequency Dependence of Signal Power and Spatial Reach of the Local Field Potential
Source: PLoS Comput Biol. 2013 Jul 18;9(7):e1003137. doi: 10.1371/journal.pcbi.1003137 (PMC3715549; doi:10.1371/journal.pcbi.1003137)
Supplement: Table S1 — Summary of the population model used for LFP simulations. Continues in Table S2. (PDF) [file pcbi.1003137.s010.pdf]

A	Model summary
**Population**	cylindrical homogeneous cortical populations
**Neuron**	passive multi-compartment neuron models
**Synapse**	current based, alpha-shaped postsynaptic current with short time constant
**Input**	uncorrelated/correlated Poisson spike train input
**Measurements**	simulated LFP

B	Population
**Type**	separate homogeneous populations consisting of N neurons<br>*Population types*: L3 pyramidal cell population, L4 stellate cell population, L5 pyramidal cell population
**Geometry**	cylinder of radius R ('cortical column') subdivided into layers (see Table S3A)
**Cell positions**	- random soma positions on a disc at soma depth z_k in vertical midpoint of corresponding cell type k, soma density $\rho = N/(\pi R^2)$<br>- random cell rotations along vertical cylindrical axis
**Parameters**	N, R, z_k, layer boundaries

C	Neuron
**Type**	passive multi-compartment neuron models with reconstructed morphologies
**Morphology**	- L3 pyramidal cell<br>- L4 stellate cell<br>- L5 pyramidal cell<br>from [71], downloaded from ModelDB, accession number 2488<br>axon compartments were removed
**Neuron dynamics**	non-spiking neurons with passive membrane with specific membrane resistance R_{m}, specific axial resistance R_{a}, and specific membrane capacitance C_{m}
**Compartments**	segments length shorter than one tenth of electrotonic length for 100 Hz resulting in 549 compartments for the L3 cell, 343 compartments for the L4 cell and 1072 compartments for the L5 cell (for chosen passive parameters and morphologies, see Table S3)
**Parameters**	R_{m}, R_{a}, C_{m}
