# Supplementary material for: Frequency Dependence of Signal Power and Spatial Reach of the Local Field Potential
Source: PLoS Comput Biol. 2013 Jul 18;9(7):e1003137. doi: 10.1371/journal.pcbi.1003137 (PMC3715549; doi:10.1371/journal.pcbi.1003137)
Supplement: Table S2 — Summary of the population model used for LFP simulations. Continued from Table S1. (PDF) [file pcbi.1003137.s011.pdf]

D	Synapse
**Type**	current synapse with α-function shaped postsynaptic currents (PSCs)
**Dynamics**	input current: $I(t) = \sum_i h(t - t_i)$<br>with kernel $h(t) = I_0 \frac{t}{\tau} e^{1-(t-t_i)/\tau} \theta(t - t_i)$<br>where t_i is the arrival time of presynaptic spike i, θ is Heaviside step function, with time constant τ and amplitude $I_0 = I_e$, I_i for excitatory and inhibitory synapses respectively
**Parameters**	τ, I_e, I_i

E	Input
	Poissonian input
**Uncorrelated input**	each cell receives n_{syn} independent excitatory inputs generated by Poissonian point processes with rate r_{syn}
**Correlated input**	each cell receives n_{syn} excitatory inputs drawn (without resampling) from a finite-sized pool (size n_{pool}) of independent Poissonian point processes with rate r_{syn} resulting in correlation $c_{\text{in}} = n_{\text{syn}}/n_{\text{pool}}$ between total input current of different cells
**Synapse placement**	synapses placed on dendrites in certain layers depending on synaptic input region (apical/homogeneous/basal) and cell type:<br>**cell type** / **apical** / **homogeneous** / **basal**<br>L3 / upper half of L23 / L1 and L23 / lower half of L23<br>L4 / - / L4 / -<br>L5 / L1 and L23 / all layers / L5 and L6<br>Random placement of synapses within allowed boundaries with uniform density with respect to membrane area (note: n_{syn} is fixed irrespective of input region)
**Parameters**	n_{syn}, r_{syn}, c_{in}

Synapse placement by cell type:

cell type	apical	homogeneous	basal
L3	upper half of L23	L1 and L23	lower half of L23
L4	-	L4	-
L5	L1 and L23	all layers	L5 and L6

F	Measurements
	Simulated LFP
**Type**	extracellular field potentials (representing the LFP) calculated using the line-source method
**Assumptions**	extracellular medium as assumed to be purely resistive (non-capacitive) and inifitely-volumed with extracellular conductivity σ_{cond}
**Electrode properties**	ideal (non-filtering) point electrode placed either in the center (r=0) of the cylindrical geometry, or offset by some distance; at depths corresponding to the middle of each layer (see Table S1:Population).
**Parameters**	σ_{cond}
