# Supplementary material for: Frequency Dependence of Signal Power and Spatial Reach of the Local Field Potential
Source: PLoS Comput Biol. 2013 Jul 18;9(7):e1003137. doi: 10.1371/journal.pcbi.1003137 (PMC3715549; doi:10.1371/journal.pcbi.1003137)
Supplement: Table S3 — Parameters of the population model used for LFP simulations. (PDF) [file pcbi.1003137.s012.pdf]

A	Population	
**Name**	**Description**	**Value**
N	number of cells	10000
R	population radius	1000 μm

z_k and layer boundaries	layer boundaries and soma positions (in units of μm in relation to cortical surface at 0 μm), derived from Stepanyants et al., Cereb Cortex 2008, 18(1):13–28:	

layer	upper boundary	soma depth z_k	lower boundary
L1	0	-	-81.6
L2/3	-81.6	-334.3	-587.1
L4	-587.1	-754.6	-922.2
L5	-922.2	-1021.1 ([1])	-1170.0
L6	-1170.0	-	-1491.7

B	Neuron	
**Name**	**Description**	**Value**
R_m	specific membrane resistance	30 kΩ·cm^2
R_a	specific axial resistance	150 Ω·cm
C_m	specific membrane capacitance	1.0 μF/cm^2

C	Synapse	
**Name**	**Description**	**Value**
τ	synaptic time constant	0.1 ms
I_e	excitatory current amplitude	50 pA

D	Input	
**Name**	**Description**	**Value**
n_syn	number of synapses	1000
r_syn	synaptic input rate	5 spikes/s
c_in	pairwise input correlation between cells	{0.01,0.1,1.0}

E	Measurements	
**Name**	**Description**	**Value**
σ_cond	extracellular conductivity	0.3 S/m

F	Simulation	
**Name**	**Description**	**Value**
dt_sim	time resolution in simulation	1/64 ms
$\mathrm{dt}_\mathrm{data}$	time resolution of data	1.0 ms
T	simulation time	either 10200 ms or 1200 ms (off-center or outside of soma layer) ([2])

([1]) the somata of the L5 cells were adjusted 25 μm upwards compared to midpoint of L5 so that apical dendrites reach L1

([2]) the first 200 ms were discarded to avoid upstart effects
